# MRPL17 is a critical regulator of mitochondrial function and a novel therapeutic target in non-small cell lung cancer

**DOI:** 10.1038/s41419-025-08343-z

**Published:** 2025-12-21

**Authors:** Chuanyong Mu, Jun-gang Zhao, Yonghua Sang, Yu-bo Yan, Ming Liu

**Affiliations:** 1 Department of Respiratory and Critical Care Medicine, First Affiliated Hospital of Soochow University, Suzhou, China; 2 Department of Thoracic Surgery, Shengjing Hospital, China Medical University, Shenyang, China; 3 Department of Cardiothoracic Surgery, The Second Affiliated Hospital of Soochow University, Suzhou, China; 4 Department of Thoracic Surgery, Harbin Medical University Cancer Hospital, Harbin, China; 5 Department of Thoracic Surgery, Shanghai Pulmonary Hospital, School of Medicine, Tongji University, Shanghai, China

**Keywords:** Non-small-cell lung cancer, Targeted therapies

## Abstract

This study investigated the role of the mitochondrial protein MRPL17 (mitochondrial ribosomal protein L17) in non-small cell lung cancer (NSCLC), exploring its expression profile, clinical significance, and therapeutic potential. Transcriptomic analyses of TCGA and single-cell RNA sequencing data revealed significant upregulation of MRPL17 in LUAD (lung adenocarcinoma) and LUSC (lung squamous cell carcinoma) tumor tissues, particularly within malignant epithelial and proliferating cancer cells. Elevated MRPL17 expression correlated with advanced stages, positive lymph node metastasis, and poorer overall survival. In vitro investigations demonstrated that silencing or knockout of MRPL17 attenuated cell viability, proliferation, migration, and invasion in NSCLC cells, while promoting apoptosis. Mechanistically, MRPL17 silencing impaired mitochondrial respiratory function, causing reduced oxygen consumption, diminished Complex I activity, and decreased ATP. These impairments were partially reversible by antioxidant treatment or glucose supplementation. Conversely, MRPL17 overexpression enhanced aggressive cellular phenotypes and mitochondrial energetic output. Bioinformatic analysis and subsequent experiments confirmed COX8A as a direct downstream target of MRPL17, mediating its pro-cancerous effects. In vivo, MRPL17 silencing suppressed NSCLC xenograft growth in nude mice, a phenomenon associated with reduced COX8A levels, mitochondrial dysfunction, heightened oxidative stress, and increased apoptosis. Thus, MRPL17 is an important pro-cancerous target in NSCLC, driving malignant progression through the regulation of mitochondrial function and cellular redox balance, with COX8A identified as a key mediator.

## Introduction

Non-small cell lung cancer (NSCLC) stands as the predominant form of lung malignancy, representing ~85% of all pulmonary carcinoma diagnoses [1]. Globally, it remains a leading cause of cancer-related mortality [2, 3], with a grim 5-year overall survival rate for advanced stages typically remaining below 20% [1]. While standard treatment modalities like surgical resection, radiation therapy, and cytotoxic chemotherapy offer some benefits, a significant portion of patients face disease recurrence or progression [4]. The inherent limitations of these conventional treatments, particularly their considerable systemic toxicities and the widespread development of acquired chemoresistance, urgently highlight the need for more precise and effective therapeutic interventions [1, 4, 5].

The therapeutic landscape for NSCLC has markedly evolved with the advent of molecularly targeted therapies and immunotherapies [5–8]. Agents such as epidermal growth factor receptor (EGFR) tyrosine kinase inhibitors (TKIs) [9], anaplastic lymphoma kinase (ALK) inhibitors [10], and programmed cell death protein 1 (PD-1)/ligand 1 (PD-L1) checkpoint inhibitors [11] have demonstrated substantial clinical benefits, including improved survival and higher objective response rates in patients with specific genomic alterations or relevant immune checkpoint expression [5–8]. However, the efficacy of these targeted approaches is frequently curtailed by the inevitable emergence of acquired resistance mechanisms (e.g., EGFR T790M mutation, ALK fusion gene rearrangements) and the intrinsic heterogeneity of tumor biology, which restricts their applicability to a subset of patients [1, 4]. This continuous challenge underscores the critical need for identifying novel, therapeutically actionable targets to overcome current limitations and expand treatment options [9, 10]. It has also been the research focus of our group [12–15].

Growing evidence robustly substantiates the pivotal role of mitochondrial hyperfunction in sustaining the aggressive proliferation, survival, and metastatic dissemination of various cancers, including NSCLC [12, 15–19]. NSCLC cells, like all other malignant cells, often exhibit profound metabolic reprogramming, characterized by heightened mitochondrial biogenesis, augmented oxidative phosphorylation (OXPHOS), and altered substrate utilization [12, 15–19]. These adaptations are essential to meet the prodigious bioenergetic and biosynthetic demands of rapid tumor growth. Given cancer’s integral reliance on robust mitochondrial activity, the targeted modulation of key mitochondrial proteins presents a compelling and innovative therapeutic paradigm for NSCLC [12, 15–19].

Among the indispensable constituents of the mitochondrial machinery, mitochondrial ribosomal protein L17 (MRPL17) is a core component of the large (39S) subunit of the mitochondrial ribosome (mitoribosome) [20–22]. It is a nuclear-encoded protein that forms an integral component of the large subunit of the mitochondrial ribosome, essential for mitochondrial protein synthesis [20–22]. Its functional role primarily involves facilitating the translation of mitochondrial DNA (mtDNA)-encoded messenger RNAs into proteins critical for OXPHOS and cellular energy production [20–22]. The mechanism of action relies on MRPL17’s integration into the ribosomal complex, where it aids in the assembly and catalytic activity of the ribosome to ensure accurate peptide chain elongation during translation [20–22]. Despite the recognized significance of mitochondrial integrity in NSCLC progression and MRPL17’s foundational role in mitochondrial functions, the role of MRPL17 within the context of human cancers, particularly NSCLC, remains largely undefined. The present study aims to comprehensively elucidate the expression profiles, functional implications, and underlying mechanisms of MRPL17 in NSCLC, thereby assessing its potential as a novel diagnostic biomarker or therapeutic target.

## Materials and methods

### Reagents and chemicals

All essential cell culture reagents, including basal media, fetal bovine serum (FBS), and a spectrum of antibiotics, were sourced from Hyclone (Logan, UT). The specific antibodies for MRPL12 and COX8A (cytochrome c oxidase subunit 8A) were acquired from Abcam (Cambridge, UK). The anti-MRPL17 antibody was obtained from Thermo Fisher Scientific (Suzhou, China). The anti-Sp1 (specificity protein 1) antibody was obtained from Cell Signaling Technology (Danvers, MA). A comprehensive array of chemical compounds, such as puromycin, polybrene, glucose and N-acetylcysteine (NAC), were procured from Sigma-Aldrich (St. Louis, MO). Fluorescence dyes were reported previously [12, 15].

### Cell and tissue acquisition

The established A549 cells, along with primary human NSCLC cells obtained from three consented patients (designated pNSCLC-1/-2/-3), and primary lung epithelial cells from two distinct donors (pEpi1-2), were acquired following the established protocols [12]. Lung adenocarcinoma (LUAD) patient samples were meticulously collected from individuals who had not undergone any prior anticancer therapeutic interventions. Prior to experimental use, all cells underwent routine screening for mycoplasma and other microbial contaminants, were authenticated via STR (Short Tandem Repeat) profiling, and subjected to rigorous morphological assessment. NSCLC tumor and adjacent normal lung tissues were obtained from consenting patients [12], with all associated human sample protocols receiving ethical sanction. All human-related procedures received full endorsement from the First affiliated Hospital of Soochow University’s Ethics Committee (SDFYY-2021-67#) and adhered strictly to the principles of the Helsinki Declaration.

### Immunohistochemistry (IHC)

Paraffin-embedded human/xenograft tissue sections underwent a series of preparatory steps: including baking, deparaffinization, and rehydration, followed by extensive washes with PBST (phosphate-buffered saline with Tween-20). To mitigate non-specific binding, sections were then blocked with 5% serum in PBST. Endogenous peroxidase activity was inactivated with hydrogen peroxide. Primary antibody incubation was conducted for 6 h at room temperature, succeeded by a 1.5-h incubation with biotin-conjugated secondary IgG antibodies. Antigen-antibody complexes were visualized using diaminobenzidine (DAB) chromogen after thorough washing.

### Quantitative real-time polymerase chain reaction (qRT-PCR)

In brief, total RNA was isolated from cell or tissue lysates utilizing TRIzol reagent, followed by reverse transcription into complementary DNA (cDNA). The amplification process was conducted in accordance with standardized methodologies [23], and *glyceraldehyde-3-phosphate dehydrogenase* (*GAPDH*) tested as the internal normalization reference. Quantitative data analysis was performed following previously validated protocols [15]. Primers specific for *MRPL17*, *MRPL12* and *COX8A* genes were obtained from Genechem (Shanghai, China).

### Western blotting

Cellular and tissue lysates underwent separation through SDS-PAGE gels ranging from 7.5 to 12.5% acrylamide concentration, followed by transfer onto polyvinylidene difluoride membranes, which were then blocked with 5% non-fat milk in PBST for 40 min, then incubated overnight at 4 °C with primary antibody solutions. After washing, membranes were incubated with horseradish peroxidase-conjugated secondary antibodies for 50 min at room temperature. Protein bands were visualized via enhanced chemiluminescence, and densitometric quantification of band intensities was performed using the ImageJ software. The uncropped blot images are included in Supplementary Fig. S1.

### Single-cell RNA sequencing

As comprehensively reported in previous studies [12, 17], single-cell RNA sequencing (scRNA-seq) data underwent rigorous computational processing utilizing Seurat v5.1.0 within the *R* statistical computing environment. This study encompassed the analysis of two distinct scRNA-seq datasets. The primary dataset comprised an integrated lung cancer dataset, which was publicly accessible from figshare (10.6084/m9.figshare.c.6222221.v3), representing a rich resource for lung cancer transcriptomics [24]. The secondary dataset, GSE131907, originated from the work of Kim et al. and was retrieved from the Gene Expression Omnibus (GEO) database [25]. Following stringent quality control measures to exclude low-quality cells and potential technical artifacts, the processed data subsequently underwent normalization employing the SCTransform method to account for technical variations. This was followed by integration utilizing rPCA (repaired principal component analysis) to harmonize data across different batches or samples. Finally, for dimensionality reduction and visualization of cellular heterogeneity, UMAP (uniform manifold approximation and projection) was applied. Cell annotations were derived directly from the original source publications of each respective dataset, ensuring consistency with established classifications.

### Gene silencing and overexpression

For gene silencing and overexpression experiments, specifically targeting human *MRPL17* or *COX8A*, lentiviral constructs were engineered. These constructs included two distinct shRNAs targeting MRPL17 (kdMRPL17-sh1-1/2), each possessing unique and verified sequences designed for robust knockdown as well as one verified shRNA targeting COX8A. Additionally, the construct encoding the MRPL17 cDNA and the COX8A cDNA were prepared for overexpression studies. These lentiviral constructs were co-transfected into HEK-293 cells along with essential lentivirus envelope constructs using Lipofectamine 3000 to produce infectious lentiviral particles. The resultant lentiviral particles, at a multiplicity of infection (MOI) of 10, were then transduced into NSCLC cells or primary human lung epithelial cells for 36 h. Afterwards, cells were maintained in complete culture medium supplemented with polybrene to enhance viral entry, and stable cell lines were established through puromycin selection over 5 passages. The efficacy of MRPL17/COX8A silencing or overexpression was rigorously validated at both the mRNA and protein levels.

### CRISPR/Cas9-mediated MRPL17 knockout (KO)

To achieve precise genetic ablation of *MRPL17*, CRISPR/Cas9-mediated knockout (KO) was performed. Initially, NSCLC cells were maintained in complete culture medium supplemented with polybrene to facilitate lentiviral transduction. These cells were then transduced with Cas9-expressing lentiviral particles [26, 27] to ensure constitutive expression of the Cas9 nuclease. Stable Cas9-expressing cells were subsequently established through rigorous puromycin selection. Two verified sgRNAs (single guide RNAs) specifically designed to target human MRPL17 were utilized, including koMRPL17-sg1 and koMRPL17-sg2. Each was cloned into a lenti-CRISPR/Cas9-KO-puro construct (Genechem, Shanghai, China). Lentiviral particles harboring the construct were then transduced into the previously established stable Cas9-expressing cells. Stable knockout cells, designated “koMRPL17”, were then carefully selected using puromycin. Through single-cell cloning, individual stable knockout cells were ultimately established, ensuring clonality. Control cells, designated “sgC” (carrying a non-targeting sgRNA), were characterized early [12, 13]. To verify the specificity of genetic manipulations, we used mitochondrial ribosomal protein L12 (MRPL12) as a negative control. Both MRPL17 and MRPL12 are essential protein components of the mitochondrial large ribosomal subunit [21]. Analysis of MRPL12 served to validate the on-target specificity of the MRPL17 manipulations.

### Cellular fluorescence staining

For cellular fluorescence staining assays, cells were inoculated into 24-well plates at a titration of 1.8 × 10^4^ cells per well in 500 µL of basal medium, followed by incubation for the pre-specified duration [16, 17, 19]. Subsequent to this incubation, cells underwent chemical fixation with 4% paraformaldehyde and exhaustive wash with PBS. Cells were then incubated with specific fluorochromes, followed by additional washes. Visualization was performed using a Leica epifluorescence microscope, with fluorescence emission intensity quantitatively measured via a Hitachi F-7000 spectrophotometer.

### Assessment of mitochondrial complex I activity and ATP levels

The enzymatic kinetics of mitochondrial complex I were precisely elucidated using a commercially available kit from Sigma [12, 17]. This methodology entailed the spectrophotometric measurement of NADH’s reversion to NAD+, a catalytic event directly mediated by complex I. A discernible attenuation in absorbance at 425 nm served as the quantitative measurement for its activity. The intracellular and tissue ATP concentrations were rigorously quantified using a commercial colorimetric kit, sourced from Sigma, in strict adherence to the manufacturer’s prescribed procedural mandates. Each individual assessment necessitated a 25 µL aliquot of either cellular or tissue homogenates (with 25 µg of total proteins).

### GSH to GSSG ratio

Quantification of the redox status, specifically the ratio between reduced glutathione (GSH) and oxidized glutathione (GSSG), was carried out employing a GSH/GSSG ratio kit procured from Thermo Fisher Scientific (Suzhou, China) [17]. Lysates were incubated with 5,5’-Dithio-bis (2-nitrobenzoic acid) (DTNB), glutathione reductase, and NADPH within a defined reaction milieu. Absorbance at 425 nm was transduced and logged over a 5–6-min temporal interval utilizing a spectrophotometer. A standard curve, delineated from authentic GSH and GSSG standards, facilitated the accurate measurement of their respective concentrations within the lysates. Each analytical determination similarly utilized 25 µL of cellular or tissue homogenates (with 25 µg of total proteins).

### Thiobarbituric acid reactive substances (TBAR) assay

A TBAR assay kit, obtained from Thermo Fisher Scientific (Suzhou, China), was deployed to rigorously test lipid peroxidation. Tissue or cellular protein lysates were allowed to react with thiobarbituric acid (TBA) to engender the TBAR complex. Subsequent to this conjugation, and following refrigeration and centrifugation to expunge any precipitates, the optical density at 535 nm was spectrophotometrically determined. Each individual assessment necessitated a 25 µL aliquot of either cellular or tissue homogenates (with 25 µg of total proteins).

### Single-stranded DNA (ssDNA) ELISA

ssDNA ELISA was conducted to detect and quantify autoantibodies targeting ssDNA. Briefly, high-binding 96-well plates were coated overnight with purified anti-ssDNA (Novus Biologicals), followed by blocking with 1% BSA in PBS-T. Diluted lysate samples and controls were then incubated for 90 min at room temperature, subsequently detected with peroxidase-conjugated anti-human IgG for 45 min. Following washes, TMB (3,3’,5,5’-Tetramethylbenzidine) substrate was added for color development, stopped with H_2_SO_4_, and absorbance was measured at 450 nm, with a reference at 620 nm.

### Transwell assays

For in vitro cell migration assays, genetically modified NSCLC cells were apportioned at a concentration of 1.3 × 10^4^ cells per well in serum-deprived medium and carefully introduced into the compartment of Transwell chambers. After a 24-h incubation period, cells that had transmigrated to the basolateral surface were immobilized, stained, and digitally imaged. In the context of in vitro cellular invasion assays, the chambers were uniformly pre-coated with Matrigel (Sigma), while all other operational paradigms remained unaltered.

### Oxygen consumption rate (OCR)

OCR was precisely delineated using an Agilent Seahorse XF24 Extracellular Flux Analyzer, rigorously adhering to previously promulgated standardized protocols [28]. Cellular respiration was comprehensively delineated by sequentially perfusing cells with specific metabolic modulators: 1 µM oligomycin, succeeded by 0.5 µM FCCP (carbonyl cyanide-p-trifluoromethoxyphenylhydrazone), and culminating with a conjoint application of 0.5 µM antimycin A and rotenone. This systematic pharmacological perturbation enabled the comprehensive quantification of basal, ATP-linked, maximal, and non-mitochondrial OCR components. All derived OCR values were normalized to the corresponding intracellular protein content.

### Additional cellular assays

A series of functional cellular assays were performed as detailed in our studies [12–14]. Genetically modified NSCLC or lung epithelial cells were seeded at a density of 3000 cells per well in 96-well plates, then incubated for a specified duration. Subsequently, a CCK-8 mixture was added, followed by an additional 90 min incubation, with absorbance measured at 450 nm using a microplate reader. Caspase-3 and -9 activity in prepared cell lysates was assessed using a Caspase-3/-9 colorimetric assay kit (Biyuntian, Wuxi, China), strictly adhering to the manufacturer’s instructions. Each treatment involved analyzing 25 µL of cellular or tissue lysates (with 25 µg total proteins). Cell death was determined by Trypan blue staining using an automatic cell counter following designated treatments.

Chromatin Immunoprecipitation (ChIP). ChIP was employed to assess the transcription factor Sp1 interacting with the *COX8A* promoter, following established protocols [29, 30]. Genomic DNA from cell lysates was cross-linked and sheared to preserve protein-DNA associations. Sp1-bound DNA fragments were isolated using an anti-Sp1 antibody. The enriched DNA was then quantified by quantitative PCR to evaluate the binding of Sp1 to the proposed *COX8A* promoter sequence (*GAGGAGGGG*, JASPAR 2024). Results were normalized to appropriate controls to provide final, standardized measurements.

### The animal xenograft studies

As reported previously [12, 14], xenograft experiments were conducted using 6-week-old nude mice (18–19 g), with an equal distribution of male and female individuals. These mice were housed at the Animal Facility at Soochow University (Suzhou, China). Six million pNSCLC-1 cells per mouse were subcutaneously (*s.c*.) injected into the flanks. Tumor dimensions were measured, and volumes were calculated using a previously described formula [26, 27]. For in situ lung cancer model, the BALB/c nude mice were anesthetized, and their chest area was prepared using the described protocols [31]. The pNSCLC-1 cell suspension, at 1 × 10^6^ cells per mouse, mixed with Matrigel, was prepared for injection. The mouse was positioned on its side [31]. Using a fine-gauge needle and a Hamilton syringe, a puncture was made through the chest wall into the right lower lung lobe. A precise volume (40 μL) of the cell suspension was slowly injected. After injection, the needle was withdrawn, the puncture site was sealed, and the incision was sutured [31]. The mouse was monitored for recovery, and tumor growth was tracked for 3 weeks. Then, lungs were surgically harvested, and the tumors were analyzed and quantified. All animal experimental protocols received ethical approval from the Institutional Animal Care and Use Committee (IACUC) and the Ethics Committee (SDFYY-2021-67#) of the First affiliated Hospital of Soochow University.

### Statistical analysis

For in vitro studies, a rigorous blinded approach was consistently employed for group allocation. These experiments were replicated across five distinct biological repetitions. Data, all demonstrating a normal distribution, were presented as mean ± standard deviation (SD). Statistical analysis was performed using SPSS version 26.0 (SPSS Co., Chicago, IL). The unpaired Student’s *t* test was utilized for comparisons between two specific groups. For comparisons involving more than two groups, one-way ANOVA with the Scheffé’s and Tukey’s test was applied. Statistical significance was defined as *P* values less than 0.05.

## Results

### TCGA analysis shows expression profile, clinicopathological associations, and prognostic and diagnostic value of *MRPL17* in NSCLC

**Fig. 1:**
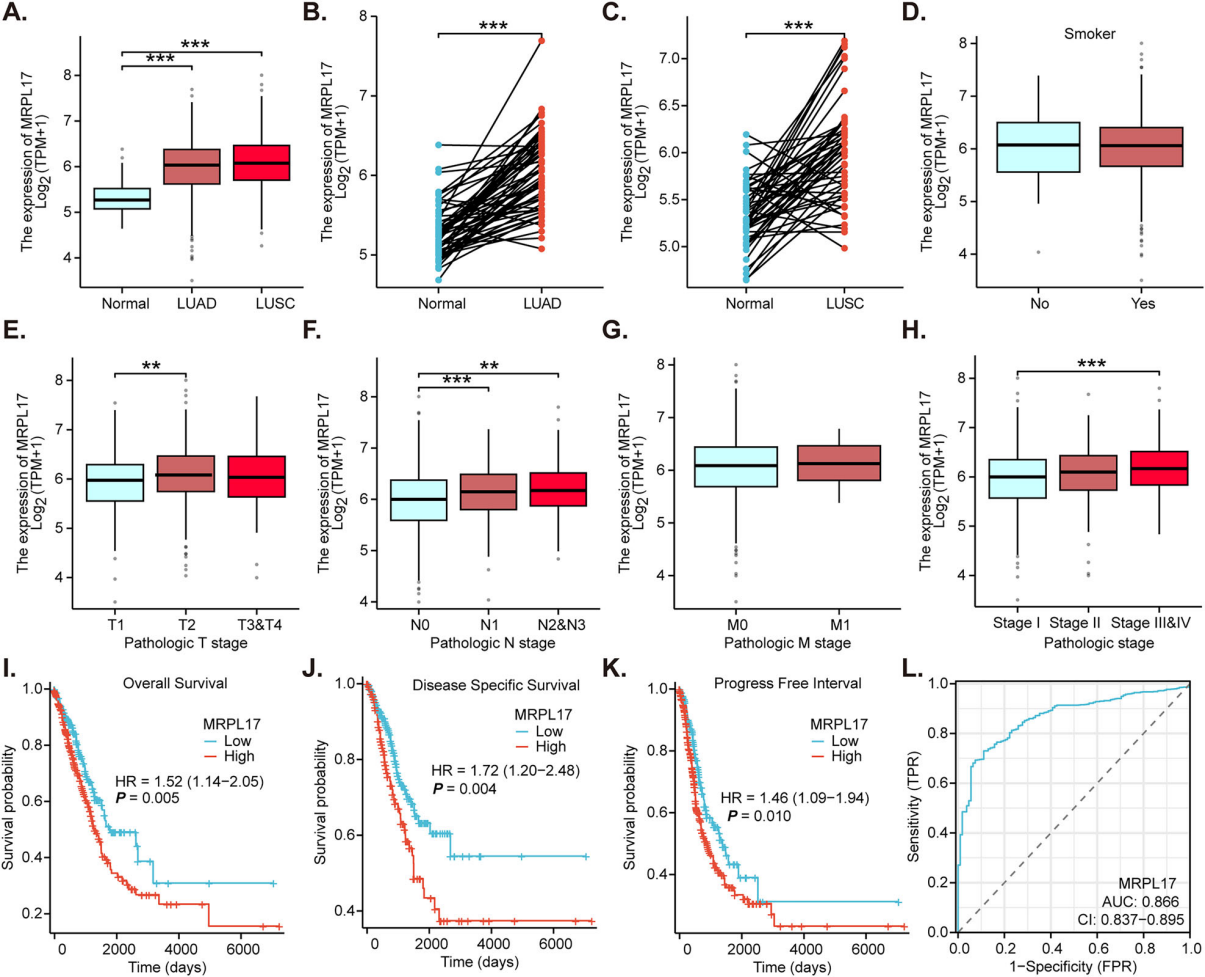
TCGA analysis shows expression profile, clinicopathological associations, and prognostic and diagnostic value of *MRPL17* in NSCLC. Transcriptomic analysis of TCGA (The Cancer Genome Atlas) datasets conducted to evaluate the expression levels of *MRPL17* in LUAD and LUSC tumor tissues compared to normal lung tissues (**A**). Direct comparison performed on paired tissue samples to assess *MRPL17* expression within LUAD (**B**) or LUSC (**C**) tumor tissues relative to adjacent normal lung tissues. The analyses were performed to investigate the potential difference in *MRPL17* expression based on the smoking status (**D**), pathological T stages (T1&T2 vs. T3&T4) (**E**), the presence or absence of lymph node metastasis (N0 vs. N1, N2&N3) (**F**), distant metastasis status (M0 vs. M1) (**G**), or across different pathological stages (Stage I vs. Stage II and Stage III&IV) (**H**) of the NSCLC patients. Kaplan-Meier survival analysis conducted to evaluate the correlation between *MRPL17* expression and overall survival (OS) (**I**), disease-specific survival (DSS) (**J**) or progression-free interval (PFI) (**K**) in NSCLC patients (combining LUSD and LUAD). Receiver operating characteristic (ROC) curve analysis performed to evaluate the diagnostic utility of *MRPL17* expression in differentiating between NSCLC and normal lung tissues (**L**). Asterisks denote statistical significance: *P < 0.05, **P < 0.01, ***P < 0.001.

Transcriptomic analysis of TCGA (The Cancer Genome Atlas) datasets revealed a statistically significant upregulation of *MRPL17* expression in both LUAD and lung squamous cell carcinoma (LUSC) tumor tissues when compared to normal lung tissues (Fig. 1A). Consistent with these findings, direct comparison of paired tissue samples further demonstrated a pronounced increase in *MRPL17* expression within LUAD tumor tissues relative to adjacent normal lung tissues (Fig. 1B). Similarly, in LUSC, *MRPL17* expression was significantly higher in tumor tissues compared to their paired normal counterparts (Fig. 1C). Collectively, these results robustly establish *MRPL17* as highly expressed in both LUAD and LUSC. Investigation into the clinical relevance of *MRPL17* expression revealed notable associations with key clinicopathological parameters. No statistically significant difference in *MRPL17* expression was observed based on the smoking status of NSCLC patients (Fig. 1D). However, *MRPL17* expression was significantly elevated in NSCLC patients presenting with advanced pathological T stages (T3&T4) compared to those with earlier stages (T1&T2) (Fig. 1E). Analogously, a marked increase in *MRPL17* expression was evident in NSCLC patients exhibiting positive lymph node metastasis (N1, N2&N3) in contrast to those without nodal involvement (N0) (Fig. 1F). While distant metastasis (M0 vs. M1) did not yield a significant difference in *MRPL17* expression (Fig. 1G), *MRPL17* levels were significantly higher in NSCLC patients with advanced pathological stages (Stage III&IV) compared to Stage I (Fig. 1H).

Kaplan-Meier survival analyses were conducted to evaluate the prognostic implications of *MRPL17* expression. A compelling association was identified, demonstrating that high expression of *MRPL17* was significantly correlated with a poorer overall survival (OS) in patients diagnosed with NSCLC (Fig. 1I, HR = 1.52, 95% CI: 1.14-2.05, *P* = 0.005). Furthermore, elevated *MRPL17* expression was independently associated with significantly worse disease-specific survival (DSS) (Fig. 1J, HR = 1.72, 95% CI: 1.20-2.48, *P* = 0.004) and a reduced progression-free interval (PFI) (Fig. 1K, HR = 1.46, 95% CI: 1.09-1.94, *P* = 0.010). These consistent findings across multiple survival endpoints underscore the predictive value of *MRPL17* expression as an indicator of an unfavorable prognosis in these specific lung cancer subtypes. To ascertain a potential diagnostic utility of *MRPL17* expression, a receiver operating characteristic (ROC) curve analysis was performed. The resulting area under the curve (AUC) was calculated to be 0.866 (95% CI: 0.837–0.895) (Fig. 1L). This strong AUC value indicates that *MRPL17* expression possesses considerable diagnostic accuracy in differentiating between tumor and normal lung tissues in NSCLC.

### Single-cell transcriptomic analysis of *MRPL17* expression, associations, and co-expression networks in lung cancer

**Fig. 2:**
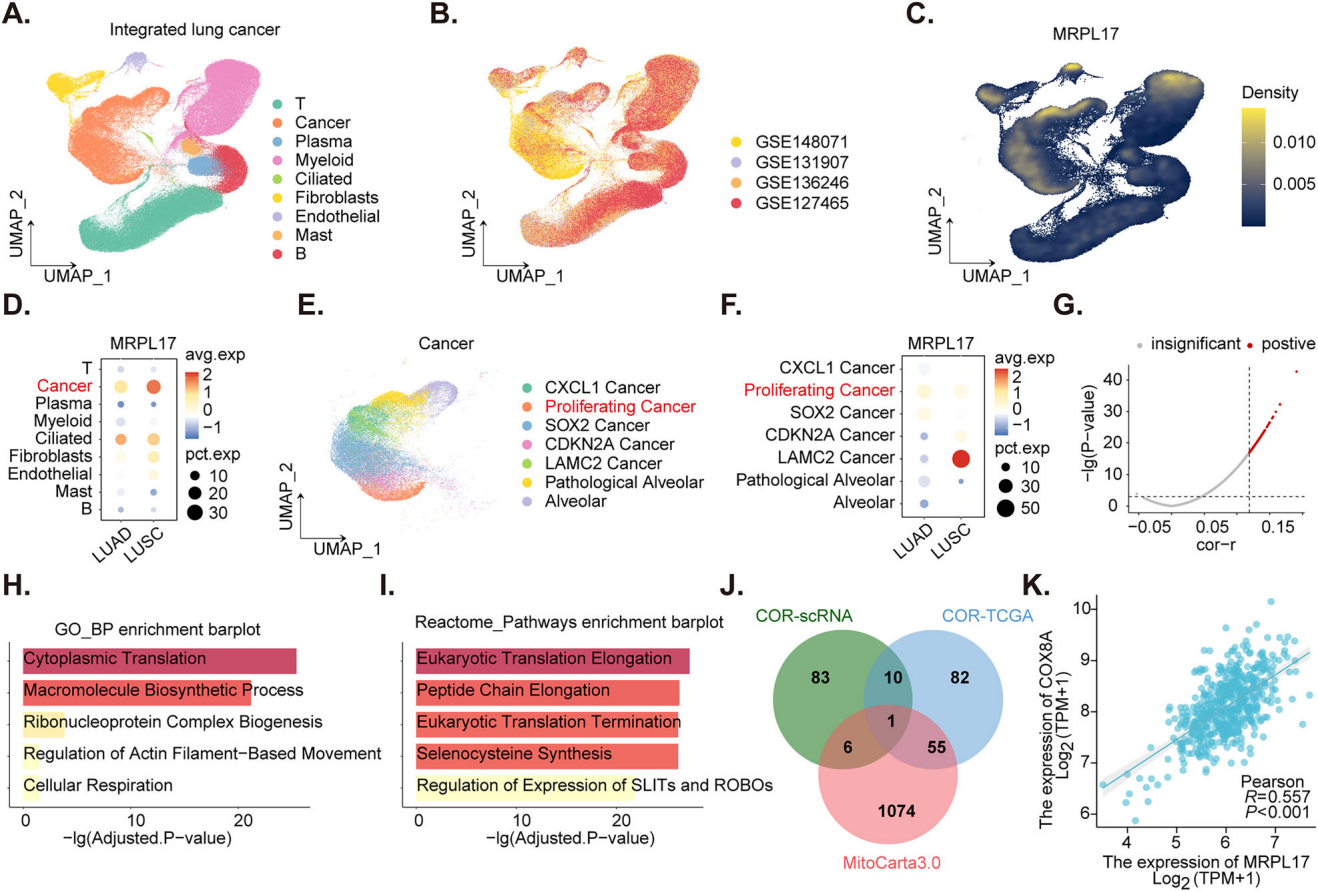
Single-cell transcriptomic analysis of *MRPL17* expression, associations, and co-expression networks in lung cancer. Dimensionality reduction plots depict cellular composition and integrated scRNA-seq data from lung cancer samples, with annotated cell populations (**A**) and displayed data sources (**B**). An expression density plot maps *MRPL17* distribution across cell types (**C**), showing predominant expression in malignant cancer cells and ciliated cells, with higher expression in both LUSC and LUAD (**D**). Cancer cell populations were sub-clustered to characterize heterogeneity (**E**), revealing *MRPL17*’s higher expression within the proliferating sub-cluster (**F**). Correlation analysis identified top 100 genes positively co-expressed (COR) with *MRPL17* in NSCLC cancer cell subpopulations (**G**). Functional and pathway enrichment analyses of these genes indicated enrichment in translation, cellular respiration, actin-mediated cell motility (**H**), and eukaryotic translation, selenocysteine synthesis, and SLIT-ROBO pathways (**I**). Comprehensive analysis using TCGA-NSCLC bulk RNA-seq data, intersected with MitoCarta3.0 and scRNA co-expressed genes in cancer cell subpopulation, identified *COX8A* as a common mitochondrial gene (**J**). The co-expression of *MRPL17* and COX8A was validated using independent TCGA data (**K**).

Dimensionality reduction plots were generated to visualize the cellular composition and integration of single-cell RNA sequencing (scRNA-seq) data from lung cancer samples. Cell populations, provided by the original authors [24], were annotated based on their unique transcriptomic profiles (Fig. 2A), and the integrated data sources were displayed, indicating the origin of each cell (Fig. 2B). An expression density plot was utilized to map the distribution of *MRPL17* expression across different cell types (Fig. 2C), revealing that *MRPL17* was predominantly expressed in malignant cancer cells and ciliated cells (Fig. 2D). Within the cancer cell population, *MRPL17* exhibited relatively higher expression in both LUSC and LUAD. Furthermore, a comparative analysis indicated that *MRPL17* overexpression was more significant in LUSC cancer cells compared to LUAD cancer cells within the NSCLC grouping (Fig. 2D). To further characterize the heterogeneity within the malignant compartment, cancer cell populations were extracted and subjected to additional sub-clustering analysis (Fig. 2E). The distribution of *MRPL17* expression across these cancer cell sub-clusters was then investigated (Fig. 2F). This analysis demonstrated that *MRPL17* exhibited higher expression levels within the proliferating sub-cluster (Fig. 2F).

Correlation analysis was performed to identify genes co-expressed with *MRPL17* within the cancer cell subpopulations in NSCLC (both LUSC and LUAD). The top 100 genes positively correlated with *MRPL17* were identified (Fig. 2G). Subsequent functional and pathway enrichment analyses were conducted on these positively correlated genes. Gene Ontology (GO) enrichment analysis revealed significant enrichment in functions related to translation, cellular respiration, and actin-mediated cell motility (Fig. 2H). Reactome pathway analysis indicated enrichment in pathways associated with eukaryotic translation elongation, peptide chain elongation, eukaryotic translation termination, selenocysteine synthesis, and the regulation of SLIT-ROBO pathways (Fig. 2I). To identify mitochondrial genes co-expressed with *MRPL17*, a comprehensive analysis was performed using TCGA-NSCLC bulk RNA-seq data. Genes with a correlation coefficient *R* > 0.5 and a *P* < 0.05 with *MRPL17* were identified as co-expressed genes. These genes were then intersected with a known list of mitochondrial genes (MitoCarta3.0) and the above scRNA co-expressed genes in cancer cell cluster (Fig. 2J). This process yielded one common gene, *COX8A* (Fig. 2J). The co-expression relationship between *MRPL17* and *COX8A* was subsequently validated using independent TCGA data, further confirming their association (Fig. 2K).

### Single-cell RNA sequencing analysis of LUAD data reveals a consistent elevation of MRPL17 expression in malignant epithelial cells observed across primary tumor sites and various metastatic locations

**Fig. 3:**
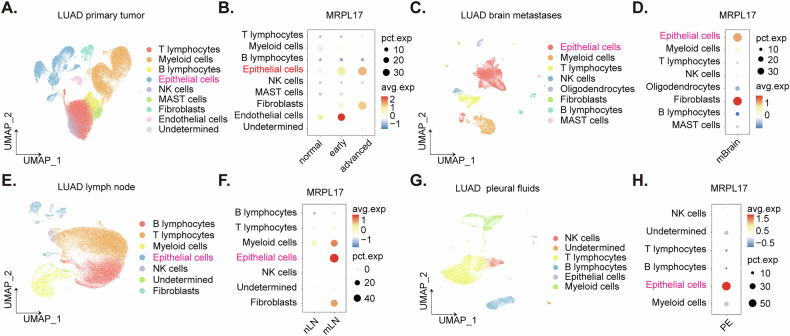
Single-cell RNA sequencing analysis of LUAD data reveals a consistent elevation of *MRPL17* expression in malignant epithelial cells observed across primary tumor sites and various metastatic locations. UMAP visualization of the primary LUAD tumor, illustrating its cellular composition by distinct cell types (**A**). A dot plot detailing the percentage of cells expressing *MRPL17* and their average expression levels across various primary tumor cell types, stratified by ‘normal,’ ‘early,’ and ‘advanced’ disease stages as contextualized by the original study (**B**). UMAP visualization of LUAD brain metastases (mBrain), depicting the distribution of various cell populations (**C**). A dot plot quantifying *MRPL17* expression within the specific cell types identified in brain metastases (**D**). UMAP visualization of LUAD lymph node metastasis (mLN), showing its cellular landscape (**E**). A dot plot presenting *MRPL17* expression within cell types found in lymph node metastasis, differentiating between non-lymph node (nLN) and metastatic lymph node (mLN) compartments (**F**). UMAP visualization of LUAD pleural effusion (PE), revealing its constituent cell types (**G**). A dot plot displaying *MRPL17* expression levels across the cellular subsets identified within pleural effusion samples (**H**).

Single-cell RNA sequencing data from a cohort of LUAD patients (GSE131907) were carefully analyzed to delineate the precise expression profiles of *MRPL17* within various cellular compartments and across different metastatic sites. Cellular identities were rigorously assigned, leveraging annotations previously established by the original authors of the dataset [25]. In the primary LUAD tumor microenvironment, as depicted in the comprehensive single-cell map (Fig. 3A), a discernible trend of increasing *MRPL17* expression was observed in malignant epithelial cells correlating with advancing disease progression (Fig. 3B), and its expression is low in epithelial cells of normal lung tissues (Fig. 3B). Furthermore, analysis of LUAD brain metastasis (mBrain) samples (Fig. 3C) revealed a prominent and high level of *MRPL17* expression specifically within the malignant epithelial cell and fibroblast populations (Fig. 3D). Investigation into LUAD lymph node metastasis (mLN) data (Fig. 3E) indicated a noteworthy elevation of *MRPL17* expression, predominantly localized within the malignant epithelial cell population (Fig. 3F). Consistent with observations in other metastatic sites, analysis of LUAD pleural effusion (PE) samples (Fig. 3G) similarly demonstrated a robust high expression of *MRPL17*, again notably concentrated within the malignant epithelial cell population (Fig. 3H). This widespread observation across various metastatic sites implies that *MRPL17* overexpression is associated with progression and metastasis of LUAD.

### MRPL17 expression is elevated in locally resected human NSCLC tissues and different NSCLC cells

**Fig. 4:**
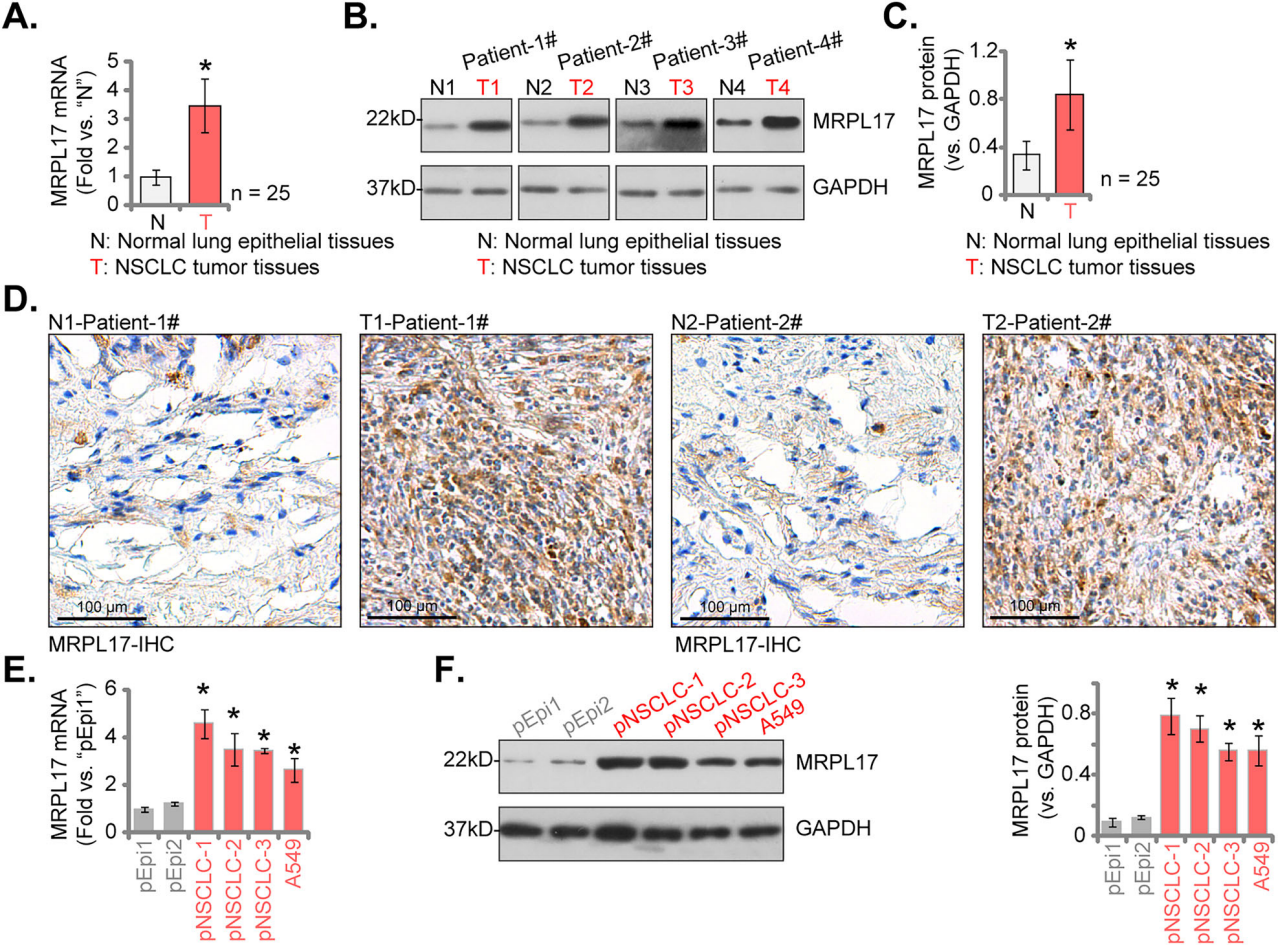
MRPL17 expression is elevated in locally resected human NSCLC tissues and different NSCLC cells. Quantitative real-time PCR (qRT-PCR) assessment of *MRPL17* mRNA transcript levels in paired NSCLC tumor (T) and adjacent non-malignant (N) lung tissues procured from a patient cohort (*n* = 25) (**A**). Representative Western blotting analyses illustrating MRPL17 protein expression in paired N and T tissues derived from four individual patients (**B**). Cohort-wide quantitative analysis based on immunoblots comparing MRPL17 protein abundance in N versus T tissues across all 25 patients (**C**). Immunohistochemical (IHC) staining demonstrating MRPL17 protein distribution and abundance in representative paired N and T tissue sections of two individual patients (**D**). Comparative qRT-PCR analysis quantifying *MRPL17* mRNA levels across various NSCLC cells (pNSCLC-1, pNSCLC-2, pNSCLC-3 primary cells and A549 cell line) relative to normal primary human lung epithelial cells (pEpi1, pEpi2) (**E**). Comparative Western blotting analysis, including quantification, assessing MRPL17 protein levels in the panel of NSCLC cells versus normal lung epithelial control cells (**F**). All quantitative data are presented as mean ± standard deviation (SD). Asterisks indicate statistical significance (*P < 0.05) relative to the N tissues or pEpi1 cells. Scale bars represent 100 µm.

We next extended our investigation by examining MRPL17 differential expression in clinical specimens and pertinent cell models. Analysis was performed on a cohort comprising 25 paired (*n* = 25) fresh NSCLC tumor specimens (“T”) and adjacent non-malignant lung tissues (“N”), surgically procured from locally resected patients at our institutions [12, 14]. qRT-PCR studies revealed a significant elevation of *MRPL17* mRNA transcript levels within the tumor tissues relative to their matched normal counterparts (Fig. 4A). Representative Western blot analyses from four individual patient pairs subsequently illustrated higher MRPL17 protein expression in tumor specimens, providing initial evidence for concordant protein upregulation (Fig. 4B). The quantitative analysis of immunoblots across the entire patient cohort (*n* = 25) confirmed that MRPL17 protein abundance was significantly augmented in NSCLC tumor tissues relative to the adjacent non-malignant controls (Fig. 4C). Furthermore, immunohistochemical (IHC) staining corroborated these findings, revealing markedly enhanced MRPL17 immunoreactivity within the neoplastic regions compared to the adjacent non-malignant pulmonary tissue in representative patient sections (Fig. 4D). We subsequently evaluated MRPL17 expression profiles in established in vitro models. Comparative analysis across multiple primary human NSCLC cells (pNSCLC-1, pNSCLC-2, pNSCLC-3, derived from three patients) and the A549 cell line, versus normal primary human lung epithelial cells (pEpi1, pEpi2 [16, 17]) indicated significantly higher *MRPL17* mRNA transcript levels in all examined NSCLC cells (Fig. 4E). Concordantly, Western blot analysis confirmed markedly elevated MRPL17 protein expression in the panel of NSCLC cells relative to the normal lung epithelial controls (Fig. 4F). These findings provide robust evidence for the significant upregulation of MRPL17 expression, at both the transcript and protein levels, in human NSCLC clinical samples and representative cells when compared to corresponding non-malignant controls.

### **MRPL17** silencing impairs NSCLC cell malignant phenotypes with minimal effects on normal lung epithelial cells

**Fig. 5:**
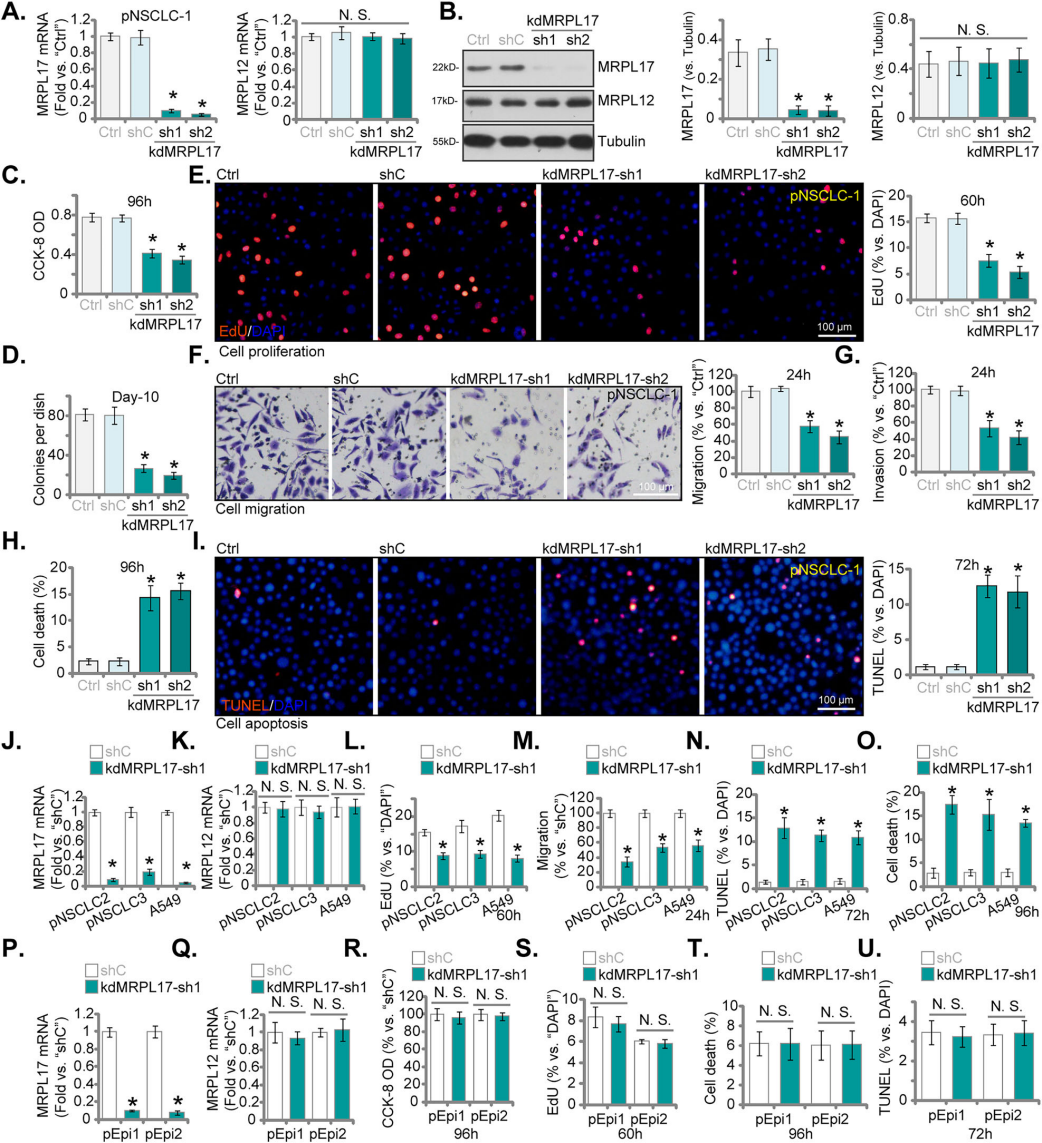
MRPL17 silencing impairs NSCLC cell malignant phenotypes with minimal effects on normal lung epithelial cells. Validation of MRPL17 silencing in pNSCLC-1 cells transduced with shC (scramble control shRNA), kdMRPL17-sh1, or kdMRPL17-sh2. Relative mRNA levels of *MRPL17* and *MRPL12* assessed by qRT-PCR (**A**). Protein abundance of MRPL17 and MRPL12 assessed by Western blot (**B**). Functional analysis of MRPL17 silencing in pNSCLC-1 cells included: determination of cell viability by CCK-8 assay (**C**); assessment of clonogenic potential by colony formation assay (**D**); measurement of cellular proliferation via nuclear EdU incorporation assay (**E**); assessment of cell migration and invasion by Transwell assays (**F**) and Matrigel Transwell (**G**) assay (G); as well as quantification of overall cell death by Trypan blue exclusion assay (**H**); and assessment of apoptosis via TUNEL staining (**I**). Analysis was extended to additional NSCLC models (pNSCLC-2, pNSCLC-3, A549) comparing kdMRPL17-sh1 to shC. Assessment included relative mRNA levels of *MRPL17* (**J**) and *MRPL12* (**K**) by qRT-PCR; measurement of cell proliferation via EdU incorporation assay (**L**); assessment of cell migration by Transwell assay (**M**); assessment of apoptosis via TUNEL assay (**N**); and quantification of overall cell death by Trypan blue exclusion assay (**O**). Comparative analysis was performed in normal primary human lung epithelial cells (pEpi1 or pEpi2) comparing kdMRPL17-sh1 to shC. Assessment included relative mRNA levels of *MRPL17* (**P**) and *MRPL12* (**Q**) by qRT-PCR; determination of cell viability by CCK-8 assay (**R**); measurement of cellular proliferation via EdU incorporation assay (**S**); quantification of overall cell death by Trypan blue exclusion assay (**T**); and assessment of apoptosis via TUNEL staining (**U**). “Ctrl” stands for the parental control cells. All quantitative data are presented as mean ± standard deviation (SD) from five independent experiments (*n* = 5). Asterisks indicate statistical significance (*P < 0.05) relative to the shC group. “N.S.” stands for non-statistical difference. Scale bars represent 100 µm.

To elucidate the functional significance of MRPL17 in NSCLC, we employed shRNA-mediated silencing in primary patient-derived NSCLC cells (pNSCLC-1). Transduction with two distinct lentiviral vectors expressing shRNAs targeting MRPL17 (kdMRPL17-sh1 and kdMRPL17-sh2, with non-overlapping sequences) caused a significant downregulation of both *MRPL17* transcript levels and protein abundance relative to control shRNA (shC) (Fig. 5A, B), whereas the expression of the control mitochondrial ribosomal protein, MRPL12, remained unaltered (Fig. 5A, B). Functionally, silencing of MRPL17 significantly attenuated key oncogenic characteristics of pNSCLC-1 cells. A marked reduction in cell viability (CCK-8 OD, Fig. 5C), clonogenic potential (Fig. 5D) and cellular proliferation, measured via nuclear EdU incorporation (Fig. 5E), was observed upon treatment with either shRNA construct compared to control shC. Furthermore, both in vitro cell migration (Fig. 5F) and invasive capacity through Matrigel (Fig. 5G) were largely inhibited following MRPL17 knockdown. Concomitantly, knockdown of MRPL17 in pNSCLC-1 cells increased overall cell death rates (tested by Trypan blue staining assays, Fig. 5H) and promoted apoptosis, as demonstrated by a significantly elevated proportion of TUNEL-positive nuclei (Fig. 5I). However, the absolute levels of cell death and apoptosis induction, while statistically significant compared to controls, generally remained below 15–20% under ese experimental conditions (Fig. 5H, I).

To corroborate these initial observations, we evaluated the consequences of MRPL17 silencing using kdMRPL17-sh1 in two other independent primary NSCLC isolates (pNSCLC-2, pNSCLC-3) and the established A549 NSCLC cell line. Robust silencing of *MRPL17* mRNA was verified in all tested models by kdMRPL17-sh1 (Fig. 5J), with *MRPL12* mRNA levels unchanged (Fig. 5K). The phenotypic outcomes mirrored those observed in pNSCLC-1 cells; specifically, MRPL17 silencing by kdMRPL17-sh1 significantly curtailed proliferation (Fig. 5L) and migration (Fig. 5M), while concurrently augmenting apoptosis induction (Fig. 5N) and overall cell death (Fig. 5O) in pNSCLC-2, pNSCLC-3, and A549 cells. Although the magnitude of these increases typically resulted in total cell death or apoptotic fractions not exceeding 15–20% across these NSCLC cells (Fig. 5N, O). To assess the potential differential dependency on MRPL17 between malignant NSCLC cells and non-malignant pulmonary epithelial cells, we investigated the impact of MRPL17 silencing (using kdMRPL17-sh1) in normal primary human lung epithelial cells (pEpi1 and pEpi2). Despite achieving efficient downregulation of *MRPL17* mRNA (but not *MRPL12*, Fig. 5P, Q), no significant alterations were observed in cell viability (Fig. 5R), cellular proliferation (Fig. 5S), overall cell death (Fig. 5T), or apoptotic indices (Fig. 5U) relative to control conditions. Collectively, these data strongly indicate a differential requirement for MRPL17, highlighting its critical role in sustaining the viability and malignant characteristics of NSCLC cells, while appearing largely dispensable for the homeostasis of normal lung epithelial cells under these experimental parameters.

### **MRPL17** silencing impairs mitochondrial function and induces oxidative stress in NSCLC cells

**Fig. 6:**
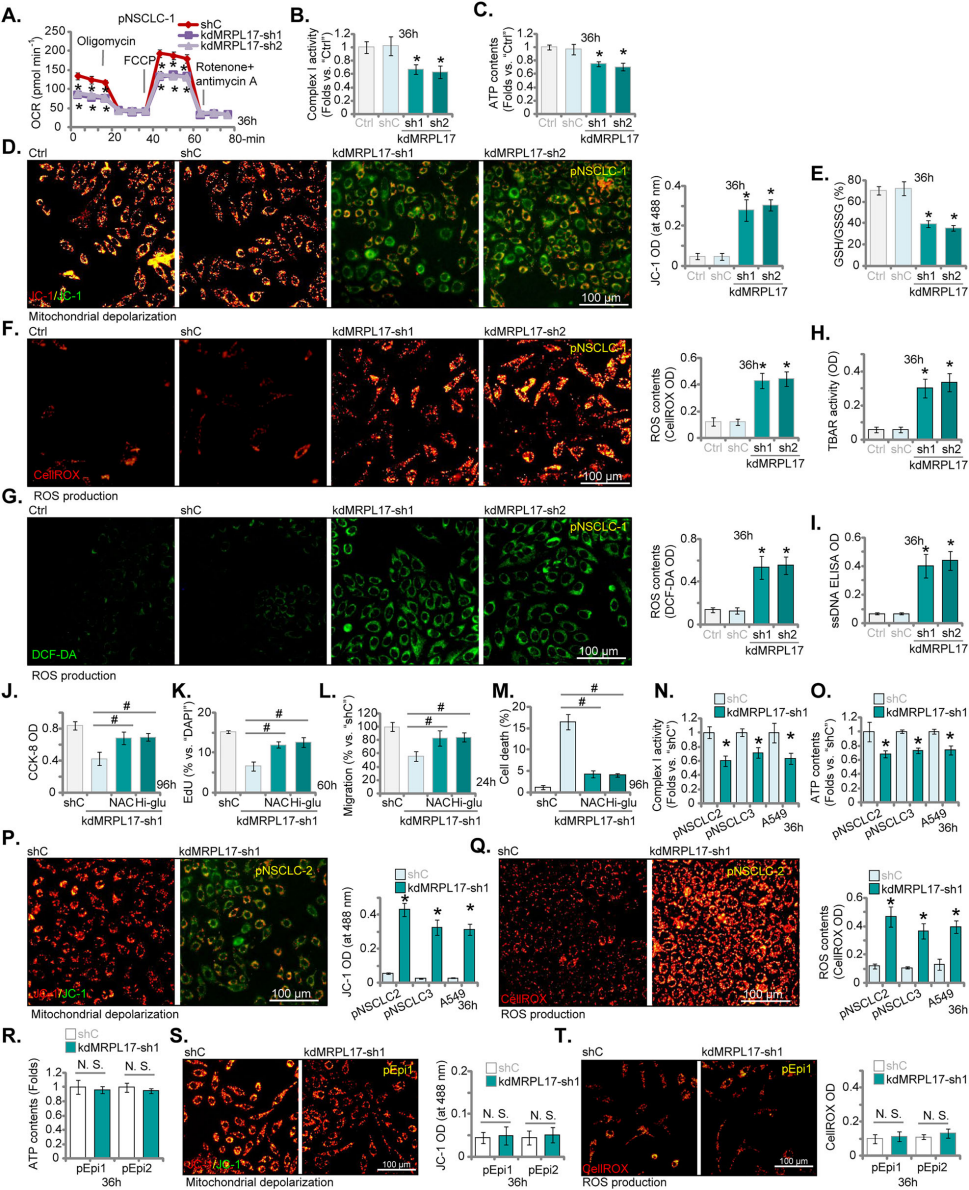
MRPL17 silencing impairs mitochondrial function and induces oxidative stress in NSCLC cells. Mitochondrial function in primary NSCLC cells (pNSCLC-1), following transduction with control shRNA (shC), kdMRPL17-sh1, or kdMRPL17-sh2, was evaluated. Assessed parameters encompassed oxygen consumption rates (OCR) determined via Seahorse metabolic analysis (**A**), enzymatic activity of mitochondrial Complex I (**B**), quantification of total cellular ATP levels (**C**), and assessment of mitochondrial membrane potential based on JC-1 monomer fluorescence intensity (**D**). Corresponding indicators of oxidative stress measured in these pNSCLC-1 cells included the cellular glutathione redox state (GSH/GSSG ratio) (**E**), intracellular reactive oxygen species (ROS) levels detected using CellROX red (**F**) and DCF-DA blue (**G**) fluorescent probes, quantification of lipid peroxidation products via TBAR assay (**H**), and measurement of single-stranded DNA (ssDNA) content via ELISA as an index of DNA damage (**I**). The potential for functional rescue was subsequently investigated in pNSCLC-1 cells, comparing kdMRPL17-sh1 expressing cells to shC controls after pre-treatment with the antioxidant N-acetylcysteine (NAC, 0.4 mM) or cultivation in high-glucose medium (Hi-glu, adding 7.5 mM); evaluated endpoints included cell viability (**J**), cellular proliferation (nuclear EdU ratio, **K**), cell migration capacity (**L**), and overall cell death levels (**M**). The mitochondrial parameters were further examined in additional NSCLC models (pNSCLC-2/3 cells, A549 cell line) comparing kdMRPL17-sh1 effect relative to shC controls, including assessment of mitochondrial Complex I activity (**N**), total cellular ATP content (**O**), mitochondrial membrane potential via JC-1 staining (**P**), and intracellular ROS production utilizing the CellROX probe (**Q**). The primary human lung epithelial cells (pEpi1 or pEpi2) expressing kdMRPL17-sh1 to shC were cultivated for 36 h, total cellular ATP content (**R**), mitochondrial membrane potential via JC-1 staining (**S**), and intracellular ROS production utilizing the CellROX probe (**T**) were tested. “Ctrl” stands for the parental control cells. All quantitative data are presented as mean ± standard deviation (SD) from five independent experiments (*n* = 5). Asterisks indicate statistical significance (*P < 0.05) relative to the shC group. # indicates *P* < 0.05 (**J**–**M**). “N.S.” stands for non-statistical difference. Scale bars represent 100 µm.

To investigate the mitochondrial consequences of silencing MRPL17, the primary NSCLC cells (pNSCLC-1) were transduced with control shRNA (shC) or shRNAs targeting MRPL17 (kdMRPL17-sh1, kdMRPL17-sh2, see Fig. 5). Silencing of MRPL17 resulted in significantly impaired mitochondrial respiratory function, characterized by reduced oxygen consumption rates (OCR) (Fig. 6A). Consistent with defective oxidative phosphorylation, mitochondrial Complex I activity (Fig. 6B) and total cellular ATP levels (Fig. 6C) were significantly decreased in MRPL17-silenced pNSCLC-1cells. Furthermore, MRPL17 knockdown induced significant mitochondrial membrane depolarization, as indicated by an increase in the JC-1 monomer fluorescence intensity (Fig. 6D). The observed mitochondrial dysfunction was associated with a significant increase in oxidative stress markers. MRPL17 silencing led to a lower cellular GSH/GSSG ratio (Fig. 6E), indicative of a shift towards an oxidized intracellular environment. Concurrently, intracellular reactive oxygen species (ROS) levels were significantly elevated, detected using both CellROX red (Fig. 6F) and DCF-DA blue probes (Fig. 6G). Further evidence of oxidative damage was provided by assessments showing increased lipid peroxidation via TBAR assay (Fig. 6H) and elevated single-stranded DNA (ssDNA) content (Fig. 6I) in MRPL17-silenced pNSCLC-1 cells.

Given the induction of oxidative stress and mitochondrial respiration disruption, we investigated whether antioxidant treatment or metabolic supplementation could alleviate the functional impairments caused by MRPL17 silencing pNSCLC-1 cells. Pre-treatment of kdMRPL17-sh1-expressing pNSCLC-1 cells with the antioxidant N-acetylcysteine (NAC) or culturing cells in higher-glucose medium (Hi-glu, adding 7.5 mM more) significantly rescued the deficits in cell viability (Fig. 6J), proliferation (Fig. 6K), and migration (Fig. 6L), and significantly attenuated the increase in cell death (Fig. 6M) observed upon MRPL17 knockdown in pNSCLC-1 cells. To determine if these mitochondrial effects were consistent across different NSCLC contexts, key functional assays were repeated in additional primary NSCLC isolates (pNSCLC-2, pNSCLC-3) and the A549 cell line using kdMRPL17-sh1. Similar to the findings in pNSCLC-1 cells, silencing of MRPL17 in these cells (see Fig. 5) resulted in significantly reduced mitochondrial Complex I activity (Fig. 6N), decreased cellular ATP content (Fig. 6O), mitochondrial depolarization assessed by JC-1 staining (Fig. 6P), and increased ROS production measured by CellROX Green (Fig. 6Q). In normal primary human lung epithelial cells (pEpi1 or pEpi2), silencing MRPL17 with the same kdMRPL17-sh1 did not alter ATP content (Fig. 6R), decrease mitochondrial membrane potential (Fig. 6S), or induce ROS production (Fig. 6T). These findings together demonstrate that MRPL17 is crucial for maintaining mitochondrial respiratory function and redox balance in NSCLC cells. Its silencing led to mitochondrial dysfunction, oxidative stress, and associated cellular impairments, which can be partially reversed by antioxidant intervention or glucose supplementation.

### MRPL17 knockout impairs NSCLC cell malignant phenotypes and mitochondrial functions

**Fig. 7:**
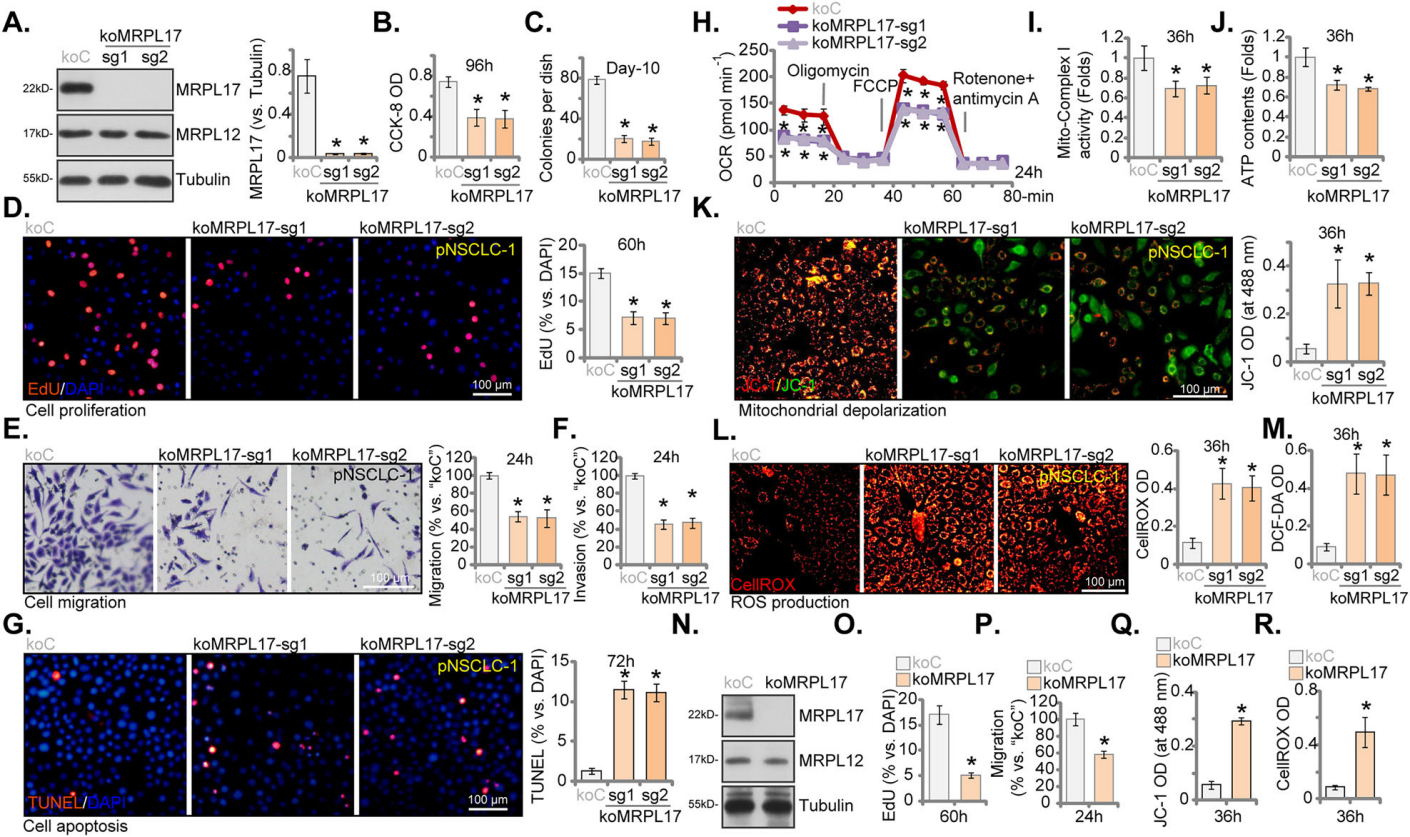
MRPL17 knockout impairs NSCLC cell malignant phenotypes and mitochondrial functions. Analysis performed in primary NSCLC cells (pNSCLC-1) comparing control cells transduced with nonsense sgRNA (koC) and two independent MRPL17 knockout clones generated using distinct sgRNAs (koMRPL17-sg1, koMRPL17-sg2). Validation assays included Western blot analysis of MRPL17 and MRPL12 protein expression (**A**). Functional assays encompassed assessment of cell viability (CCK-8 OD, **B**), evaluation of clonogenic potential via colony formation assay (**C**), measurement of cellular proliferation via nuclear EdU incorporation (**D**), determination of cell migration (**E**) and invasion (**F**) capacities using Transwell assays, and quantification of apoptosis via nuclear TUNEL staining assay (**G**). Assessment of mitochondrial homeostasis involved measurement of oxygen consumption rates (OCR) (**H**), determination of mitochondrial Complex I enzymatic activity (**I**), quantification of total cellular ATP content (**J**), analysis of mitochondrial membrane potential via JC-1 staining (**K**), and detection of intracellular ROS levels using CellROX (**L**) and DCF-DA (**M**) fluorescent indicators. Experiments were also conducted in another primary NSCLC cell isolate (pNSCLC-2 cells) comparing control (koC) to MRPL17 knockout (koMRPL17, using koMRPL17-sg1) cells; these included Western blot verification of MRPL17 protein depletion (**N**), assessment of cellular proliferation via EdU assay (**O**), determination of cell migration via Transwell assay (**P**), analysis of mitochondrial membrane potential via JC-1 staining (**Q**), and measurement of intracellular ROS generation using CellROX (**R**). All quantitative data are presented as mean ± standard deviation (SD) from five independent experiments (n = 5). Asterisks indicate statistical significance (*P < 0.05) relative to the koC group. Scale bars represent 100 µm.

To definitively establish the functional role of MRPL17 in NSCLC cells and mitigate concerns regarding potential shRNA off-target effects, we employed CRISPR/Cas9-mediated gene editing to generate MRPL17 knockout (koMRPL17) primary NSCLC cells (pNSCLC-1). Effective targeting with two independent single guide RNAs (sgRNAs, koMRPL17-sg1, koMRPL17-sg2) caused complete ablation of MRPL17 protein expression, verified by Western blot analysis (Fig. 7A), while the abundance of the control mitochondrial ribosomal protein MRPL12 was unaltered (Fig. 7A). Genetic ablation of MRPL17 precipitated significant functional deficits. Relative to the control cells with nonsense sgRNA (koC), both independent MRPL17 KO clones manifested compromised cell viability (Fig. 7B), attenuated clonogenic potential (Fig. 7C), and curtailed cellular proliferation, evidenced by reduced EdU incorporation (Fig. 7D). Moreover, MRPL17 KO profoundly suppressed both migratory (Fig. 7E) and invasive capacities of pNSCLC-1 cells (Fig. 7F). Concomitantly, loss of MRPL17 significantly promoted apoptosis, quantified via nuclear TUNEL staining assay (Fig. 7G). Subsequently, the consequences of MRPL17 ablation for mitochondrial homeostasis in pNSCLC-1 cells were studied. MRPL17 KO cells displayed markedly perturbed mitochondrial respiration, manifesting as significantly reduced basal and maximal OCR (Fig. 7H). These respiratory deficiencies were accompanied by significantly diminished mitochondrial Complex I enzymatic activity (Fig. 7I) and reduced total cellular ATP contents (Fig. 7J). Furthermore, genetic deletion of MRPL17 caused significant mitochondrial membrane depolarization, ascertained via JC-1 staining analysis (Fig. 7K). Concomitant with this mitochondrial dysregulation, MRPL17 KO engendered substantial oxidative stress and significantly elevated intracellular ROS levels, detected independently using CellROX (Fig. 7L) and DCF-DA (Fig. 7M) fluorescent indicators.

To corroborate the broader relevance of these findings, MRPL17 was genetically ablated (using koMRPL17-sg1) in an independent primary NSCLC cell isolate (pNSCLC-2). Verification of successful MRPL17 protein depletion was obtained via Western blot (Fig. 7N). The phenotypic consequences largely recapitulated those observed in pNSCLC-1 cells; specifically, MRPL17 KO in pNSCLC-2 cells significantly curtailed cellular proliferation (Fig. 7O) and suppressed cell migration (Fig. 7P). Moreover, these MRPL17-null cells exhibited significant mitochondrial depolarization (JC-1 monomer intensity increasing, Fig. 7Q) and elevated intracellular ROS generation (CellROX intensity increasing, Fig. 7R), thereby substantiating the integral role of MRPL17 in preserving mitochondrial integrity and redox equilibrium across diverse primary NSCLC cells.

### Ectopic overexpression of MRPL17 correlates causes enhanced NSCLC cellular phenotype and augmented mitochondrial functions

**Fig. 8:**
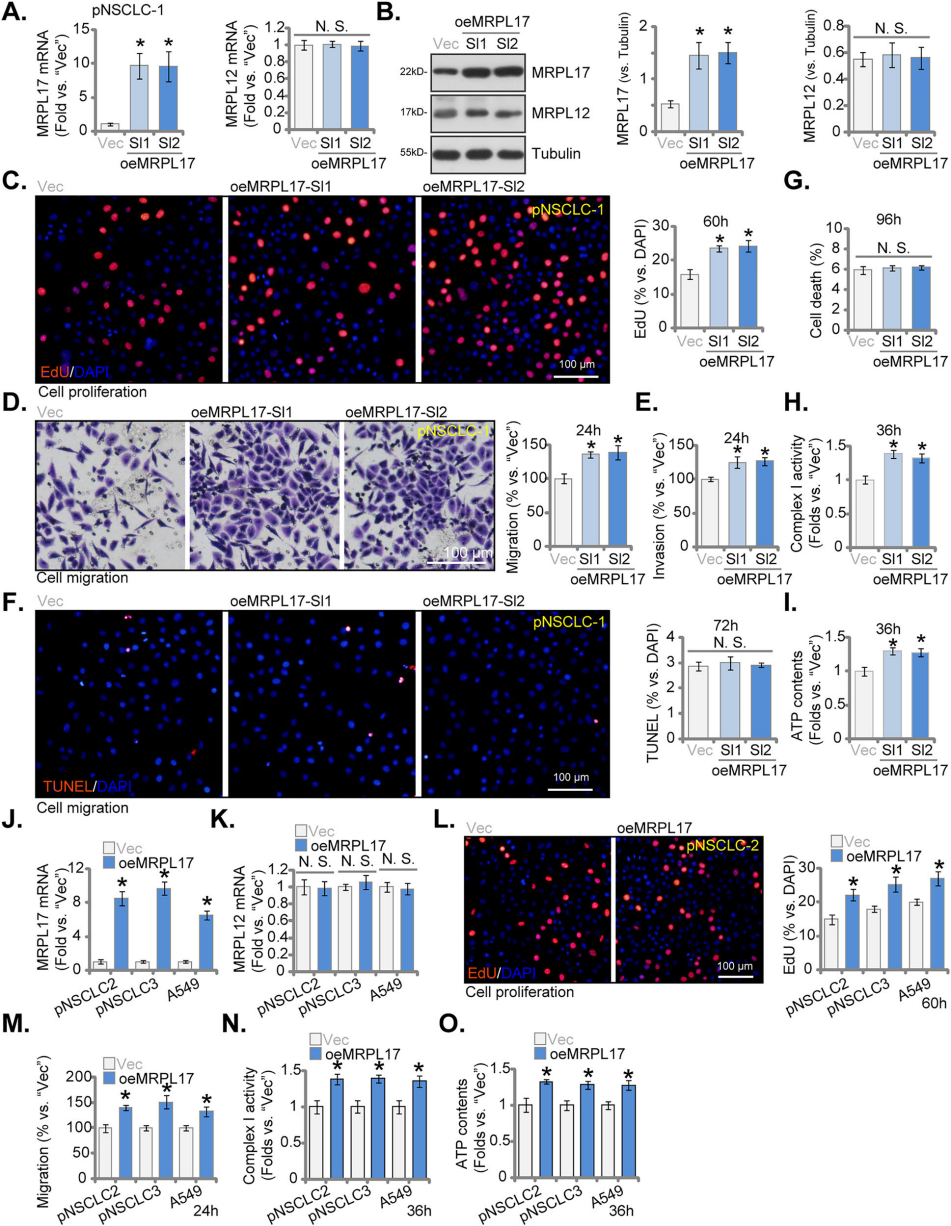
Ectopic overexpression of MRPL17 correlates causes enhanced NSCLC cellular phenotype and augmented mitochondrial functions. Analysis performed in primary NSCLC cells (pNSCLC-1) comparing empty vector control (Vec) or two independent stable cell colonies overexpressing MRPL17 (oeMRPL17-Sl1, oeMRPL17-Sl2). Validation included qRT-PCR analysis of *MRPL17* and *MRPL12* mRNA transcript levels (**A**) and Western blot assessment of MRPL17 and MRPL12 protein abundance (**B**). Functional characterization encompassed assessment of cellular proliferation via nuclear EdU incorporation assays (**C**), evaluation of cell migration (**D**) and invasion (**E**) capacities using Transwell assays, examination of apoptosis via nuclear TUNEL staining assay (**F**), and determination of overall cell death rates via Trypan blue staining assay (**G**). Mitochondrial activity assessment involved measurement of Complex I enzymatic activity (**H**) and quantification of total cellular ATP content (**I**). Analysis was extended to additional primary NSCLC isolates (pNSCLC-2, pNSCLC-3) and the A549 cell line (Vec vs oeMRPL17), including qRT-PCR validation of *MRPL17* (**J**) and *MRPL12* (**K**) mRNA levels, assessment of cellular proliferation via EdU assay (**L**), determination of cell migration via Transwell assay (**M**), measurement of Complex I activity (**N**), and quantification of total cellular ATP levels (**O**). All quantitative data are presented as mean ± standard deviation (SD) from five independent experiments (n = 5). Asterisks indicate statistical significance (*P < 0.05) relative to the Vec group. “N.S.” stands for non-statistical difference. Scale bars represent 100 µm.

To complement the above shRNA/KO studies and examine the effects of increased MRPL17 expression, we employed a lentiviral vector to stably overexpress MRPL17 in primary NSCLC cells (pNSCLC-1). Two independent stable cell colonies (designated oeMRPL17-Sl1 and oeMRPL17-Sl2) were established. Successful overexpression was confirmed by significantly elevated *MRPL17* mRNA transcripts (Fig. 8A) and increased MRPL17 protein abundance (Fig. 8B) compared to cells transduced with an empty vector control (Vec). This overexpression was specific, as the levels of the control MRPL12 remained unchanged at both the mRNA (Fig. 8A) and protein levels (Fig. 8B). Functionally, elevated expression of MRPL17 promoted aggressive cellular phenotypes in pNSCLC-1 cells. Compared to vector control cells, both oeMRPL17-Sl1 and oeMRPL17-Sl2 pNSCLC-1 cell colonies exhibited significantly increased rates of cellular proliferation, as determined by nuclear EdU incorporation assays (Fig. 8C). Furthermore, MRPL17 overexpression significantly enhanced cell migration (Fig. 8D) and invasion through Matrigel (Fig. 8E), tested via Transwell assays. Overexpression of MRPL17 did not significantly alter the basal rates of pNSCLC-1 cell apoptosis (nuclear TUNEL staining assay, Fig. 8F) or overall cell death (Fig. 8G). Consistent with the observed enhancement of cellular functions requiring significant energy supply, we assessed key parameters of mitochondrial activity. Overexpression of MRPL17 in pNSCLC-1 cells led to a significant increase in mitochondrial Complex I enzymatic activity (Fig. 8H) and elevated total cellular ATP content (Fig. 8I) compared to control cells. To ascertain the broader applicability of these overexpression effects, MRPL17 was overexpressed in additional primary NSCLC cell isolates (pNSCLC-2, pNSCLC-3) and the A549 cell line. Specific and significant overexpression of *MRPL17* mRNA (but not *MRPL12*) was confirmed in these cell models (Fig. 8J, K). In concordance with the findings in pNSCLC-1 cells, ectopic MRPL17 overexpression significantly enhanced cellular proliferation (Fig. 8L), cell migration (Fig. 8M), mitochondrial Complex I activity (Fig. 8N), and total cellular ATP levels (Fig. 8O) across pNSCLC-2, pNSCLC-3, and A549 cells. These overexpression experiments collectively indicate that heightened expression of MRPL17 correlates with an enhanced NSCLC cellular phenotype and augmented mitochondrial functions.

### COX8A serves as a downstream effector of MRPL17, mediating its pro-cancerous effects in pNSCLC-1 cells

**Fig. 9:**
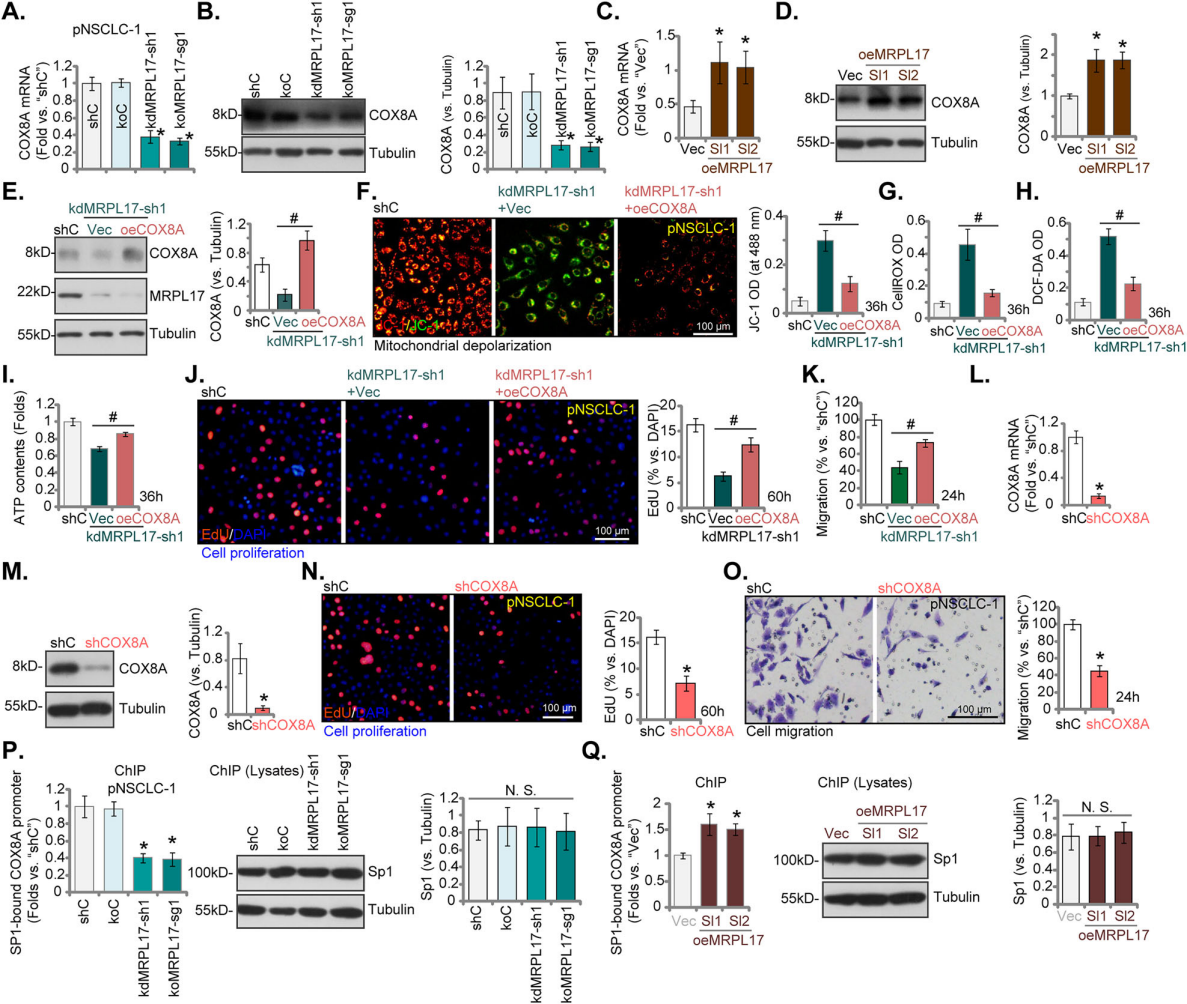
COX8A serves as a downstream effector of MRPL17, mediating its pro-cancerous effects in pNSCLC-1 cells. Expression of *COX8A* mRNA and protein levels in pNSCLC-1 cells with shC (scramble control shRNA), kdMRPL17-sh1, nonsense sgRNA (koC) or MRPL17 knockout using koMRPL17-sg1, empty vector control (Vec) or two independent stable cell colonies overexpressing MRPL17 (oeMRPL17-Sl1, oeMRPL17-Sl2) were tested (**A**–**D**); In MRPL17-silenced cells (shMRPL17-sh1), the expression of COX8A and endogenous MRPL17 was examined subsequent to ectopic overexpression of COX8A (oeCOX8A) (**E**). Functional characterization encompassed assessment of mitochondrial membrane potential based on JC-1 monomer fluorescence intensity (**F**), intracellular reactive oxygen species (ROS) levels detected using CellROX red (**G**) and DCF-DA blue (**H**) fluorescent probes, and quantification of total cellular ATP levels (**I**), as well as assessment of cellular proliferation via nuclear EdU incorporation assays (**J**) and evaluation of cell migration using Transwell assays (**K**). *COX8A* mRNA and protein levels were determined in pNSCLC-1 cells stably transduced with shC or COX8A specific shRNA (shCOX8A) (**L**, **M**). Cellular proliferation (**N**) and migration (**O**) were evaluated as well. Chromatin immunoprecipitation (ChIP) assays measured the binding of the transcription factor **Sp1** to the *COX8B* promoter in pNSCLC-1 cells with altered MRPL17 expression (shRNA-mediated knockdown, CRISRP/Cas9-induced knockout, or overexpression). The data were normalized (**P**, **Q**). Additionally, the total protein levels of Sp1 were analyzed from cell lysates to confirm its expression across all experimental conditions (**P**, **Q**). All quantitative data are presented as mean ± standard deviation (SD) from five independent experiments (*n* = 5). Asterisks indicate statistical significance (*P < 0.05) relative to the shC or Vec group. # indicates *P* < 0.05 (**E**–**K**). “N.S.” stands for non-statistical difference. Scale bars represent 100 µm.

Bioinformatic analyses consistently identified *COX8A* as a pivotal mitochondrial gene exhibiting strong association with *MRPL17* in NSCLC cells (Fig. 2). Thus, experiments were carried out to elucidate whether COX8A functions as a downstream target of MRPL17 and contributes to its pro-oncogenic properties. As depicted in Fig. 9A, B, MRPL17 silencing (achieved using shMRPL17-sh1, see Figs. 5 and 6) or KO (via shMRPL17-sg1, see Fig. 7) resulted in a statistically significant reduction in both mRNA and protein levels of COX8A within pNSCLC-1 cells. Conversely, the enforced overexpression of MRPL17 (oeMRPL17-Sl1 and oeMRPL17-Sl2, see Fig. 8) precipitated a notable elevation in *COX8A* mRNA and protein expression (Fig. 9C, D). These findings robustly indicate that MRPL17 exerts a positive regulatory influence on COX8A expression. Subsequently, we investigated whether the reintroduction of COX8A could functionally abrogate the consequences of MRPL17 depletion. Our observations revealed that the ectopic overexpression of COX8A (oeCOX8A) in MRPL17-silencing cells (using shMRPL17-sh1) successfully restored COX8A protein expression, but did not alter endogenous MRPL17 protein levels (Fig. 9E). COX8A overexpression significantly inhibited shMRPL17-sh1-induced mitochondrial functional impairment in pNSCLC-1 cells. Specifically, COX8A overexpression inhibited shMRPL17-sh1-induced mitochondrial depolarization, which was characterized by the accumulation of JC-1 green monomers (Fig. 9F). It also reduced the increase in intracellular ROS generation, as measured by CellROX and DCF-DA intensity (Fig. 9G, H), and restored ATP levels that had been reduced by shMRPL17-sh1 in pNSCLC-1 cells (Fig. 9I). Functionally, the impaired cellular proliferation and the attenuated cell migration induced by MRPL17 knockdown were partly rescued by COX8A overexpression (Fig. 9J, K), thereby demonstrating that COX8A acts as a crucial downstream target of MRPL17’s pro-cancerous effects.

To further corroborate the independent role of COX8A in pNSCLC-1 cell proliferation and migration, we performed direct genetic silencing of COX8A. As anticipated, the knockdown of COX8A, using shCOX8A, efficiently diminished both the mRNA and protein levels of COX8A (Fig. 9L, M). Importantly, COX8A silencing significantly suppressed both cellular proliferation (Fig. 9N) and cellular migration (Fig. 9O) in pNSCLC-1 cells, thereby recapitulating the phenotypic outcomes observed following MRPL17 knockdown. In culmination, the findings support that COX8A is a direct downstream target of MRPL17, essential for mediating its pro-cancerous effects on cellular proliferation and migration in pNSCLC-1 cells.

MRPL17 regulates COX8A at both the mRNA and protein levels in NSCLC cells, suggesting a potential transcriptional mechanism (Fig. 9A–D). This led us to test whether MRPL17 influences the binding of Sp1, a known transcription factor for COX8A [32]. Using a ChIP assay, we found that silencing (using shMRPL17-sh1) or knocking out (via shMRPL17-sg1) MRPL17 significantly impaired Sp1’s ability to bind to the COX8A promoter in pNSCLC-1 cells (Fig. 9P). Conversely, overexpressing MRPL17 increased Sp1 binding to COX8A promoter region (Fig. 9Q). Critically, these effects were not due to changes in the total amount of Sp1 protein (Fig. 9P, Q). These findings suggest that MRPL17 likely regulates the transcriptional activity of COX8A by modulating the binding of Sp1 to its promoter.

### Silencing of MRPL17 attenuates subcutaneous and in situ NSCLC xenograft tumor growth in nude mice

**Fig. 10:**
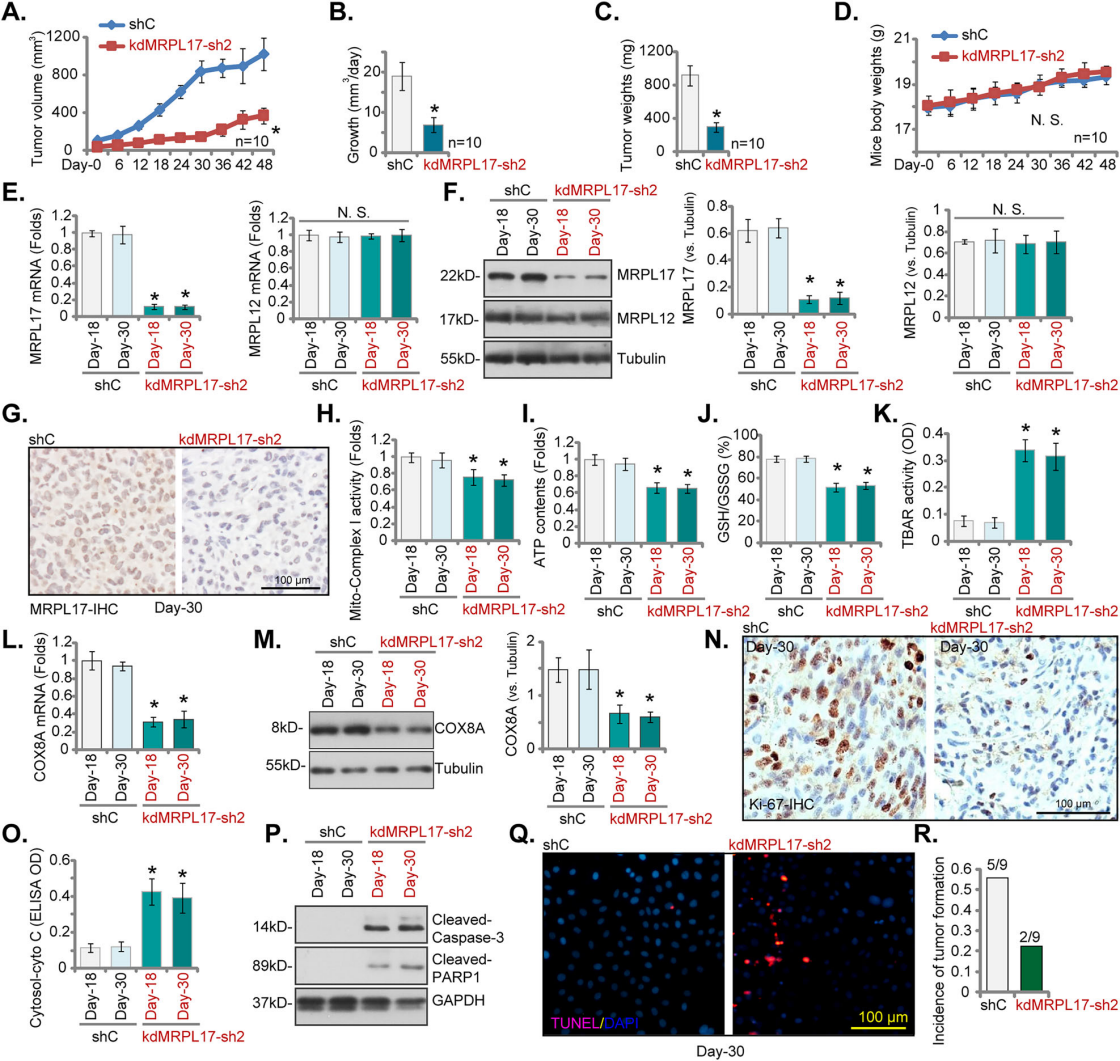
Silencing of MRPL17 attenuates subcutaneous and in situ NSCLC xenograft tumor growth in nude mice. Analysis of subcutaneous xenografts derived from primary pNSCLC-1 cells stably expressing control shRNA (shC) or shRNA targeting MRPL17 (kdMRPL17-sh2) engrafted into nude mice. Longitudinal monitoring of tumor volume over 48 days post-initiation (Day-0 defined as 3 weeks post-engraftment) (**A**), calculation of the derived tumor growth rate (**B**), assessment of explanted tumor mass at Day-48 (**C**), and longitudinal monitoring of host body weights (**D**). Validation of target modulation within tumor tissues explanted at Day-18 and Day-30 included qRT-PCR analysis of *MRPL17*, *MRPL12* and *COX8A* mRNA levels (**E**, **L**), immunoblotting analysis with quantification of MRPL17, MRPL12 and COX8A protein levels (**F**, **M**), and immunohistochemical (IHC) assessment of MRPL17 protein expression at Day-30 (**G**). Analyses of mitochondrial function and redox state performed on lysates from tumors explanted at Day-18 and Day-30 encompassed measurement of mitochondrial Complex I enzymatic activity (**H**), quantification of total cellular ATP contents (**I**), determination of the cellular GSH/GSSG ratio (**J**), and measurement of lipid peroxidation via TBAR assay (**K**). Further assessments included immunohistochemical evaluation of Ki-67-positive proliferating cells in tumor sections (**N**), quantification of cytosolic Cytochrome c levels in tumor lysates (**O**), immunoblotting analysis of Cleaved-Caspase-3 and Cleaved PARP1 protein levels in tumor lysates (**P**), and TUNEL assay for detection of apoptotic cells in tumor sections (**Q**). The bar graph illustrated the incidence of tumor formation, defined as the proportion of mice that successfully formed clear in situ pNSCLC-1 tumors in both the shC control group and the kdMRPL17-sh2 group. A significant decrease in in situ pNSCLC-1 tumor formation was observed in the kdMRPL17-sh2 group (2 out of 9 mice) compared to the shC control group (5 out of 9 mice) (**R**). Statistical significance was assessed relative to the shC control group (*P* < 0.05 denoted by asterisks; N.S., non-significant, *P* > 0.05). Data for **A**–**D** reflect *n* = 10 mice per group. For **E**–**Q** analyses utilized five distinct tissues randomly selected per xenograft (*n* = 5). For **R**, *n* = 9 mice per group. Scale bars represent 100 µm.

To evaluate the in vivo tumorigenicity and growth dynamics following MRPL17 silencing, the primary pNSCLC-1 cells stably expressing control shRNA (shC) or kdMRPL17-sh2 (see Figs. 4–5) were subcutaneously engrafted into nude mice. Longitudinal tumor growth monitoring was initiated 3 weeks post-engraftment (Day-0). Xenografts derived from MRPL17-silenced cells manifested significantly retarded growth kinetics relative to control tumors, culminating in substantially diminished tumor volumes throughout the 48-day observation period (Fig. 10A) and a markedly reduced tumor growth rate (Fig. 10B). Accordingly, tumors explanted at the study endpoint (Day-48) from the kdMRPL17-sh2 cohort exhibited significantly reduced mass compared to those from the shC control cohort (Fig. 10C). Notably, this pronounced anti-tumorigenic effect was achieved without discernible systemic toxicity, as evidenced by comparable host body weight trajectories between the experimental groups (Fig. 10D). The analyses of tumor tissues explanted at intermediate time points (Day-18 and Day-30) verified persistent and specific downregulation of MRPL17 expression within the kdMRPL17-sh2 group xenografts. Significantly diminished levels of *MRPL17* mRNA transcripts (Fig. 10E) and MRPL17 protein, assessed by immunoblotting and immunohistochemistry (Fig. 10F, G), were confirmed relative to shC controls. Conversely, the expression of the control mitochondrial ribosomal protein MRPL12 remained unperturbed at both the transcript and protein levels (Fig. 10E, F). Functionally, mirroring the in vitro observations, explanted tumors from the MRPL17-silenced group manifested significant mitochondrial impairments, including reduced Complex I enzymatic activity (Fig. 10H) and lower total ATP content (Fig. 10I) at both Day-18 and Day-30. Moreover, these tumors exhibited clear indices of heightened oxidative stress, specifically a decreased GSH/GSSG ratio (Fig. 10J) and elevated lipid peroxidation, determined via TBAR assay (Fig. 10K). The mRNA and protein levels of COX8A were also significantly decreased in the kdMRPL17-sh2 group xenograft tissues (Fig. 10L, M).

IHC evaluation demonstrated a significantly reduced fraction of Ki-67-positive proliferating cells within kdMRPL17-sh2 tumors compared to controls at Day 30 (Fig. 10N). Concomitant analyses revealed multiple indicators of enhanced apoptosis in MRPL17-depleted tumors. These included significantly elevated levels of cytosolic Cytochrome c, signifying mitochondrial outer membrane permeabilization (Fig. 10O), coupled with increased abundance of the executioner markers Cleaved-Caspase-3 and Cleaved PARP1 (Fig. 10P). Enhanced apoptotic cell death within the kdMRPL17-sh2 xenografts was further substantiated by TUNEL assay results (Fig. 10Q). Collectively, these in vivo findings rigorously demonstrate that MRPL17 silencing potently suppressed NSCLC tumor progression, a phenomenon mechanistically linked to compromised mitochondrial function, heightened oxidative stress, downregulated COX8A expression, diminished cell proliferation, and augmented apoptotic cell death within the tumor tissues. Experiments were also carried out to determine the role of MRPL17 in promoting in situ lung cancer growth. The quantified results showed that in the shC control group, in situ pNSCLC-1 xenografts were successfully established in 5 of 9 mice, whereas only 2 of 9 mice in the kdMRPL17-sh2 xenograft group developed clear in situ xenografts (Fig. 10R). These results suggest that silencing MRPL17 significantly reduced pNSCLC-1 cells’ ability to grow and form in situ xenografts in the lung.

## Discussion

Identifying novel therapeutic targets for NSCLC remained critically important due to the pervasive challenges of drug resistance and limited efficacy of existing treatments, particularly in advanced disease stages [33–35]. The intricate interplay between tumor metabolism and progression underscored the continuous need to elucidate molecular vulnerabilities that could be exploited for more effective therapeutic strategies [33–35]. Mitochondrial protein dysregulation is a frequent occurrence in NSCLC, significantly contributing to its aggressive phenotype and progression [15–19]. ADCK2 (AarF domain containing kinase 2), a mitochondrial enzyme critical for coenzyme Q biosynthesis and fatty acid metabolism [36], is consistently overexpressed in NSCLC tissues, correlating with diminished patient overall survival [19]. Experimental suppression of ADCK2 profoundly inhibited NSCLC cell viability, proliferation, and motility while simultaneously inducing apoptosis, disrupting core mitochondrial functions, and attenuating Akt-mTOR signaling, ultimately suppressing xenograft growth in murine models [19]. Similarly, the inner mitochondrial membrane protease YME1L (YME1 Like 1 ATPase) is demonstrably upregulated in NSCLC [18]. Its genetic silencing or knockout abrogated NSCLC cell proliferation and migration, induced apoptosis and mitochondrial dysfunction, and suppressed xenograft growth in vivo [18]. Furthermore, POLRMT (RNA polymerase mitochondrial), an enzyme vital for mtDNA transcription and thus integral to mitochondrial protein synthesis and cellular energy production, is overexpressed and demonstrably critical for NSCLC cell growth both in vitro and in vivo [15]. Our recent studies have shown that mitochondrial carrier homolog 2 (MTCH2) is consistently overexpressed in NSCLC, correlating with poor patient prognosis, and its functional perturbation significantly impaired NSCLC cell proliferation, migration, and invasion, inducing apoptosis and profoundly disrupting mitochondrial function [12]. One recent study also identifies TIMM23 (translocase of inner mitochondrial membrane 23) as a novel pro-tumorigenic factor in NSCLC, demonstrating that its overexpression promotes tumor growth and progression by maintaining mitochondrial hyperfunction [16]. NDUFS8 (NADH:ubiquinone oxidoreductase core subunit S8), a crucial subunit of mitochondrial complex I, is overexpressed in NSCLC and promotes tumor growth, proliferation, and radio-resistance by enhancing mitochondrial function and Akt-mTOR signaling [17].

Our comprehensive investigation strongly supported MRPL17 as a significant novel target in NSCLC. The consistent upregulation of MRPL17 in NSCLC tissues, as revealed by extensive transcriptomic and single-cell RNA sequencing analyses as well as in locally resected cancer tissues and various NSCLC cell types, pointed to its pervasive involvement in NSCLC pathogenesis. Its elevated expression, specifically within malignant epithelial cells and proliferating cancer cell sub-clusters, aligned with its observed correlation with advanced pathological stages and positive lymph node metastasis, which are established indicators of aggressive disease. More importantly, the direct association between high *MRPL17* expression and poorer overall survival in NSCLC patients underscored its potential as a robust prognostic biomarker, warranting further clinical validation.

The functional studies further solidified MRPL17’s role as a pro-oncogenic factor. In vitro investigations unequivocally demonstrated that the silencing or genetic KO of MRPL17 in NSCLC cells significantly attenuated cell viability, proliferation, migration, and invasion, concurrently promoting apoptosis and overall cell death. Crucially, these detrimental effects were minimal in normal lung epithelial cells, highlighting a differential dependency, suggesting a favorable therapeutic potential. Mechanistically, our findings revealed that MRPL17 silencing impaired mitochondrial respiratory function, characterized by reduced oxygen consumption rates, diminished Complex I activity, and decreased ATP levels, concurrently inducing mitochondrial membrane depolarization and significant oxidative stress. These cellular impairments were partially reversible by antioxidant treatment or glucose supplementation. Conversely, MRPL17 overexpression enhanced aggressive cellular phenotypes and augmented mitochondrial energetic output, confirming its central role in supporting the metabolic demands of proliferating cancer cells. The in vivo xenograft models robustly demonstrated that MRPL17 silencing significantly suppressed NSCLC tumor growth, corroborating the in vitro observations and reinforcing its therapeutic potential. Silencing the MRPL17 significantly also reduced the ability of lung cancer cells to grow and form in situ tumors in a mouse model.

COX8A, identified as a key mitochondrial gene co-expressed with MRPL17 in our bioinformatic analysis, played a crucial role in the mitochondrial electron transport chain as a component of Complex IV [37, 38]. While its direct involvement in human cancer had been less extensively characterized compared to other mitochondrial proteins, evidence suggested that dysregulation of mitochondrial oxidative phosphorylation components, including COX subunits, could profoundly influence cancer cell metabolism, growth, and survival [15, 17, 39, 40]. Our study demonstrated that MRPL17-promoted NSCLC cell malignant phenotypes were, at least in part, mediated via the regulation of COX8A expression. MRPL17 silencing or knockout decreased *COX8A* mRNA and protein levels in pNSCLC-1 cells, while overexpression increased them; additionally, reintroducing COX8A partially rescued the impaired proliferation and migration caused by MRPL17 silencing. The ChIP assay results imply that MRPL17 regulates the transcriptional activity of COX8A by modulating the binding of the Sp1 transcription factor to its promoter. Importantly, shRNA-induced silencing of COX8A inhibited pNSCLC-1 cell proliferation and migration. The observed reduction in COX8A levels in pNSCLC-1 xenograft tumors upon MRPL17 silencing in vivo, coupled with concomitant mitochondrial dysfunction, oxidative stress, decreased proliferation, and increased apoptosis, collectively supported a mechanism where MRPL17 sustained mitochondrial hyper-function necessary for NSCLC progression by regulating key components of the respiratory chain, with COX8A being a possible key mediator.

In conclusion, this study comprehensively established MRPL17 as a pivotal pro-cancerous target in NSCLC. Its overexpression is not only a prognostic indicator for poor patient outcomes but also functionally drives malignant progression by precisely regulating mitochondrial function and cellular metabolism, with COX8A serving as a key effector. While the inherent challenge of targeting mitochondrial proteins is recognized, several strategies present viable avenues for the future development of MRPL17-directed therapies in NSCLC. The challenge of targeting this protein can be addressed through modern strategies, including the use of high-resolution structural data to design highly selective small-molecule inhibitors. This approach can be combined with advanced delivery systems that overcome biological barriers and reduce systemic toxicity. Lastly, our findings from the genetic knockout and silencing experiments provide a strong basis for the exploration of gene-targeted therapies. The use of nucleic acid-based drugs, such as small interfering RNAs (siRNAs) or antisense oligonucleotides (ASOs), could offer a direct and highly specific method to downregulate MRPL17 expression within cancerous cells and the development of nanoparticle delivery systems for these gene therapies is a rapidly evolving field. All these will pave the way for a new class of targeted treatments of NSCLC.

## Supplementary information

Original data

Data Set

## Data Availability

All data are included in the Figures and Supplementary Materials.
