# Supplementary figures and images for: MRPL17 is a critical regulator of mitochondrial function and a novel therapeutic target in non-small cell lung cancer
Source: Cell Death Dis. 2025 Dec 21;17(1):105. doi: 10.1038/s41419-025-08343-z (PMC12848032; doi:10.1038/s41419-025-08343-z)

**Figure 4.**

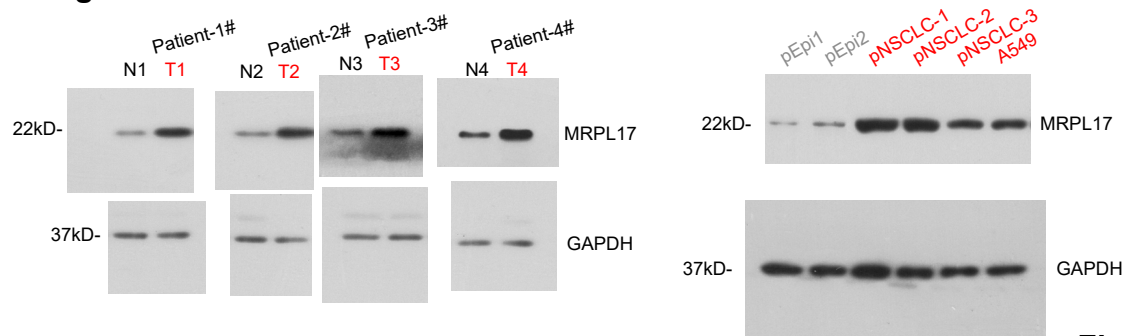

**Figure 8.**

**Figure 5.**

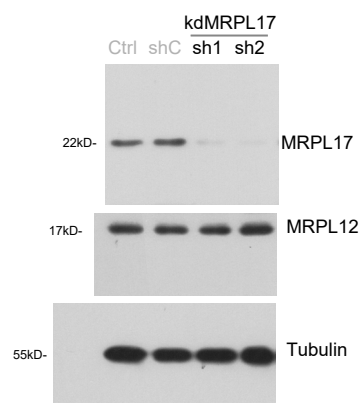

**Figure 7.**

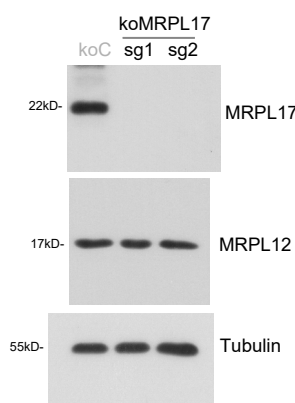

**Figure 10.**

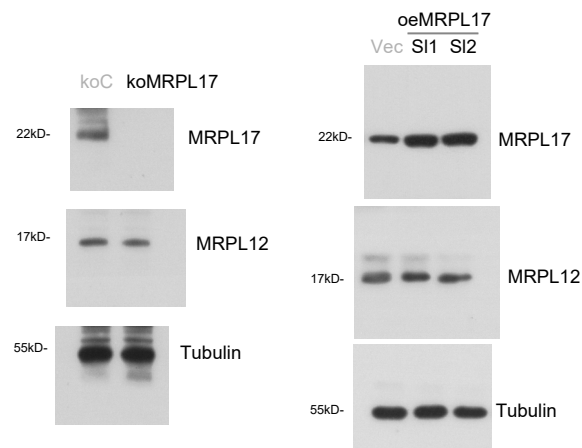

**Figure 9.**

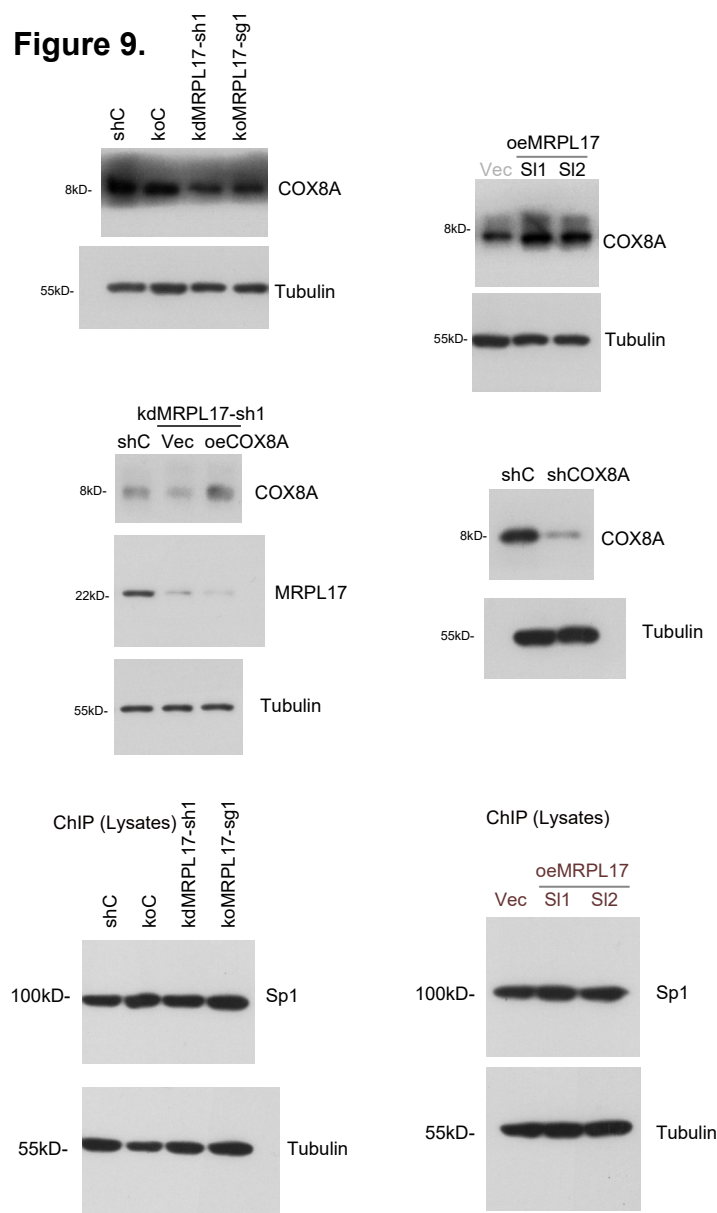

**Figure S1. The uncropped blotting images.**

Supplement: Supplementary file 1 — Original data [file 41419_2025_8343_MOESM1_ESM.pdf]
